# Migraine, comorbidity, and risks of severe maternal and neonatal morbidity or mortality: a population-based cohort study

**DOI:** 10.1093/aje/kwag008

**Published:** 2026-01-15

**Authors:** Carmela Melina Albanese, Susan J Bondy, Christine Lay, Manav V Vyas, Zhiyin Li, Jun Guan, Hilary K Brown

**Affiliations:** Dalla Lana School of Public Health, University of Toronto, Toronto, ON, Canada; ICES, Toronto, ON, Canada; Dalla Lana School of Public Health, University of Toronto, Toronto, ON, Canada; ICES, Toronto, ON, Canada; Centre for Headache, Women’s College Hospital, Toronto, ON, Canada; Centre for Headache, Women’s College Hospital, Toronto, ON, Canada; Li Ka Shing Knowledge Institute, St. Michael’s Hospital-Unity Health Toronto, Toronto, ON Canada; ICES, Toronto, ON, Canada; ICES, Toronto, ON, Canada; Dalla Lana School of Public Health, University of Toronto, Toronto, ON, Canada; ICES, Toronto, ON, Canada; Department of Health and Society, University of Toronto Scarborough, Toronto, ON, Canada

**Keywords:** migraine disorders, comorbidity, pregnancy complications, maternal mortality, perinatal mortality, routinely collected health data, Ontario, Canada

## Abstract

Migraine is a neurological disease associated with adverse perinatal outcomes. We examined the separate and combined impacts of migraine and comorbidity on risks of severe maternal morbidity/mortality (SMM-M) and severe neonatal morbidity/mortality (SNM-M). This population-based cohort study of pregnancies (*n* = 2 643 335) in Ontario, Canada, 2007-2022, compared females with (1) pre-pregnancy migraine and ≥1 other chronic conditions, (2) migraine alone, (3) other chronic conditions alone, and (4) neither migraine/other chronic conditions (referent) using modified Poisson regression. Attributable proportion due to interaction (aAP) reflected additive interaction between migraine and comorbidity. In the cohort, 6.8% had migraine and other chronic conditions, 3.2% migraine alone, 45.7% other chronic conditions alone, and 44.3% neither. The incidence of SMM-M was 1.6%, while SNM-M affected 7.1% of neonates. Risks of SMM-M and SNM-M were greatest in those doubly exposed (aRR_SMM-M_ 1.60, 95% CI 1.54-1.66; aRR_SNM-M_ 1.43, 1.39-1.46), followed by other chronic conditions alone (aRR_SMM-M_ 1.34, 1.32-1.37; aRR_SNM-M_ 1.30, 1.28-1.32), and migraine alone (aRR_SMM-M_ 1.13, 1.07-1.20; aRR_SNM-M_ 1.07, 1.04-1.11). Additive interaction was small for SMM-M (aAP 7.4%, 2.2-12.5) and SNM-M (aAP 3.7%, 0.3-6.9). Although synergistic effects were small, findings suggest individuals with migraine and comorbidity could benefit from preconception and perinatal supports to reduce their risks of perinatal complications.

## Introduction

Migraine is a neurological disease affecting 1-in-5 reproductive-aged women.^1^ Migraine features include unilateral headaches, vision loss, sensitivity to light and/or sound, and nausea/vomiting; additional neurological symptoms (aura) include blind spots, pins and needles, and speech disturbances.^2^ Migraine is a leading cause of disability^3^ and is associated with maternal and neonatal complications, including stroke,^4^ preeclampsia, preterm birth, and low birthweight.^5^ The mechanisms underlying these associations are unclear, but may include vascular epiphenomena among individuals with migraine^6^^,^^7^ and inflammation.^8^ Medications used as acute^9^^,^^10^ and preventive^11^ migraine treatments might also contribute. These factors, combined with changes in vasculature that occur during pregnancy, nature’s “stress-test,”^12^ may explain the risks of maternal and neonatal complications associated with migraine.

Comorbidity, defined as the presence of disease in addition to an index condition,^13^ is common alongside migraine.^14^ Migraine-associated comorbidities affect autoimmune, cardiovascular, cerebrovascular, gastrointestinal, metabolic, neurodevelopmental, neurologic, psychiatric, and respiratory systems, and also include sleep disorders and non-migraine chronic pain syndromes.^14^^,^^15^ The plethora of comorbidities that co-occur alongside migraine can be explained by shared pathophysiological mechanisms, including cortical excitability (chronic pain, metabolic, neurologic, psychiatric, sleep-related, vascular conditions), inflammation (autoimmune, chronic pain, gastrointestinal, metabolic, respiratory conditions), sensitization (chronic pain), endothelial activation (cardio/cerebrovascular disease), and altered neurotransmission (neurodevelopmental, psychiatric, sleep disorders);^14^^,^^15^ genetic and environmental factors; and, in certain cases, bi-directional relationships between migraine and other chronic conditions.^14^

Despite emerging research showing that over two-thirds of pregnant people with migraine also experience comorbidity (Albanese CM, et al.; Dalla Lana School of Public Health, University of Toronto; unpublished data; 2025), only a handful of studies have examined risks associated with migraine and comorbidity in pregnancy. Findings from the Omega Study in the United States reported higher risks of hypertensive disorders of pregnancy among individuals with migraine and comorbid mood disorders, asthma, and obesity, and among those with migraine alone, compared to those with neither condition.^16^^-^^18^ The separate and combined impacts of migraine and comorbidity on other perinatal outcomes, including severe maternal morbidity or mortality (SMM-M) and severe neonatal morbidity or mortality (SNM-M)—critical indicators of serious maternal and neonatal complications, remain unknown.

Understanding the relationships between migraine, comorbidity, and maternal and neonatal complications is important to improve healthcare strategies for individuals with migraine to optimize their health outcomes. Therefore, we conducted a population-based retrospective cohort study to examine the separate and combined effects of pre-pregnancy migraine and comorbidity on the risks of SMM-M and SNM-M.

## Methods

### Study design and setting

This population-based retrospective cohort study utilized health administrative data from Ontario, Canada. Under the Ontario Health Insurance Plan (OHIP), residents have universal health insurance, which covers medically necessary physician and hospital care. Health administrative datasets were analyzed at ICES, which is an independent, non-profit research institute whose legal status under Ontario’s health information privacy laws allows it to collect and analyze healthcare and demographic data, without consent, for health system evaluation and improvement. This study followed RECORD^19^ guidelines and was approved by the University of Toronto’s research ethics board (#43867).

### Data sources

Several databases were accessed at ICES (Table S1). At ICES, databases are deterministically linked using a unique encoded identifier.^20^ Validation of ICES datasets found a high degree of accuracy with re-abstraction in coded demographic data, the top 50 most responsible diagnoses, and most procedures, including procedures related to childbirth (>90% accuracy).^21^ Perinatal data in the Canadian Institute for Health Information Discharge Abstract Database (CIHI-DAD) are accurate for most pregnancy-associated outcomes.^22^

The data used in this study are not available for replication via the authors. The dataset from this study is held securely in coded form at ICES.

### Participants

Documented pregnancies (Table S2) conceived between April 1, 2007, and March 31, 2022, to females aged 13-54 years were included. Only those with ≥2 years of continuous OHIP coverage before conception were eligible. Analyses examining SNM-M were restricted to singleton pregnancies ending in a livebirth.

### Variables and measurement

Pre-pregnancy history of migraine was defined as ≥1 physician visits (OHIP 346) or ≥1 emergency department visits or hospital admissions (*ICD-10*: G43) with a migraine diagnostic code within 5 years before conception (Table S3). This algorithm has specificity of 93.5%, sensitivity of 26.5%, and 81.7% agreement with self-reported migraine diagnosis.^23^ In additional analyses, we required ≥1 physician visits or ≥1 emergency department visits or hospital admissions and a lifetime lookback period from birth or entry into OHIP to identify migraine (specificity: 84.8%, sensitivity: 49.1%, 78.5% agreement).^23^ While more sensitive than the main definition, the latter definition may capture individuals with migraine activity less proximal to pregnancy.

Thirty-five chronic conditions empirically or theoretically associated with migraine, informed by prior literature^14^^,^^15^ and clinical input, were measured using validated algorithms or disease registries at ICES (Table S4) applied to the 5 years before conception.

Our outcomes were SMM-M and SNM-M, important indicators used by the Canadian Perinatal Surveillance System (CPSS) to monitor perinatal health in Canada.^24^ The definition of SMM was based on an algorithm (Table S5) developed by the CPSS and later adapted^25^ that encompasses 40 diagnoses and procedures indicating life-threatening pregnancy-related conditions characterized by prolonged hospitalization or high case-fatality rate—important indicators of “near-miss” scenarios. SMM-M was defined as any SMM or death from any cause from conception to 42 days postpartum. Our definition of SNM was based on CPSS’ Canadian version of the Neonatal Adverse Outcomes Indicator (NAOI), a validated measure that includes 14 newborn complications and 7 procedures associated with neonatal death and hospital readmission in the first year of life^26^ (Table S5). We additionally included birthweight <1500 g and birth before 32 weeks gestation as indicators of SNM, because of their relevance in the study of migraine and other chronic conditions in pregnancy and their inclusion in other countries’ (ie, Australia, United Kingdom, France) validated NAOIs.^27^^-^^29^ SNM-M was defined as any SNM or death from any cause within 28 days after birth, or death before hospital discharge.

Confounders controlled for in the analysis were year of conception, maternal age, parity, neighborhood-level income quintile, rural/remote residence, immigrant/refugee status (each defined at conception), and recent history of interpersonal violence (<2 years prior to conception). In SNM-M analyses, we additionally adjusted for newborn sex (Table S6).

The framework illustrating our conceptualization of relationships between key variables in the association between migraine and study outcomes builds on Misra’s Integrated Perinatal Health Framework^30^ and is presented in Figure S1.

### Statistical analyses

First, we compared sociodemographic and reproductive health characteristics among individuals with both migraine and other chronic conditions, migraine alone, and other chronic conditions alone to individuals with neither migraine nor other chronic conditions using standardized differences.^31^

Next, modified Poisson regression with generalized estimating equations^32^ (by mother’s unique identification number to account for clustering of more than one pregnancy to the same individual) was used to compare the risks of SMM-M and SNM-M in individuals with (1) both pre-pregnancy migraine and other chronic conditions, (2) migraine alone, and (3) other chronic conditions alone to those with neither pre-pregnancy migraine nor other chronic conditions. We estimated both unadjusted and adjusted relative risks (RR) and corresponding 95% CIs. Predicted probabilities of SMM-M and SNM-M were estimated for each exposure group as follows: for the migraine alone group, we exponentiated the sum of the intercept value and the beta coefficient for the independent variable “migraine”; for the other chronic condition alone group, we exponentiated the sum of the intercept value and the beta coefficient for the independent variable “other chronic conditions”; for the doubly exposed group, we obtained the predicted probability of the outcome by exponentiating the sum of the intercept, beta coefficients for the independent variables indicating the presence of “migraine” and “other chronic conditions,” and the beta coefficient for the term corresponding to the “migraine x other chronic condition” interaction. Covariates were set at their reference values.

We then examined potential synergistic effects of migraine and other chronic conditions by calculating two indicators of additive interaction. First, we calculated relative excess risk due to interaction (RERI), which measures whether additive interaction is positive, negative, or zero using relative risks: RR_11_ − RR_10_ − RR_01_ + 1, where RERI > 0 indicates positive interaction between migraine and other chronic conditions.^33^ We also estimated attributable proportion due to interaction (AP), which measures the proportion of *excess* risk in the doubly exposed group that is due to the interaction, calculated as RERI/RR_11._^33^ We calculated 95% CI using the MOVER method.^34^

Finally, using known values of sensitivity and specificity for the migraine algorithm,^23^ we conducted a simple quantitative bias analysis to determine the bias-adjusted association between migraine and the outcomes using a spreadsheet created by Fox et al.^35^

As missing data were minimal (<0.4% for covariates), we conducted a complete-case analysis, including in the analytic cohort only pregnancies with non-missing data on covariates. Analyses were conducted in SAS Enterprise Guide 8.3 (SAS Institute, Cary, NC, USA)^36^ and R version 4.2.0.^37^

In additional analyses, we repeated the main analyses: (1) utilizing the migraine algorithm with a lifetime lookback period and (2) comparing those with a migraine diagnostic code during the index pregnancy to those without a migraine diagnostic code during pregnancy. We also examined physical and psychiatric chronic conditions separately, as well as chronic conditions grouped by body system. We additionally examined individual indicators of SMM-M and SNM-M as separate outcomes. We repeated analyses examining SMM-M in pregnancies ending in a livebirth or stillbirth, which allowed us to include multiple pregnancy status as a covariate. Among individuals who were born in Ontario in 1988 and later and therefore had data available during childhood, we further adjusted for adverse childhood experiences associated with migraine and adverse perinatal outcomes.^38^^,^^39^

## Results

### Cohort characteristics

We identified 2 643 335 pregnancies to 1 357 043 individuals during the study period. After excluding 9500 pregnancies (0.4%) with missing covariate data (specifically, income quintile or rural/remote residence) the analytic cohort comprised 2 633 835 pregnancies to 1 354 549 individuals. Among the included pregnancies, 6.8% were to individuals who had migraine and other chronic conditions, 3.2% migraine alone, 45.7% other chronic conditions alone, and 44.3% no migraine nor other chronic conditions. Compared to those without migraine or other chronic conditions, individuals with both migraine and other chronic conditions were more likely to be multiparous, have a pregnancy ending in a miscarriage, live in the lowest neighborhood income quintile, and have histories of recent interpersonal violence and adverse childhood experiences, but were less likely to have a pregnancy ending in a live birth and to be immigrants. Individuals with migraine alone were similar in sociodemographic characteristics to those with neither migraine nor other chronic conditions, while individuals with other chronic conditions alone were more likely to be multiparous and have histories of recent interpersonal violence and adverse childhood experiences (Table S7).

### Main findings

**Figure 1:**
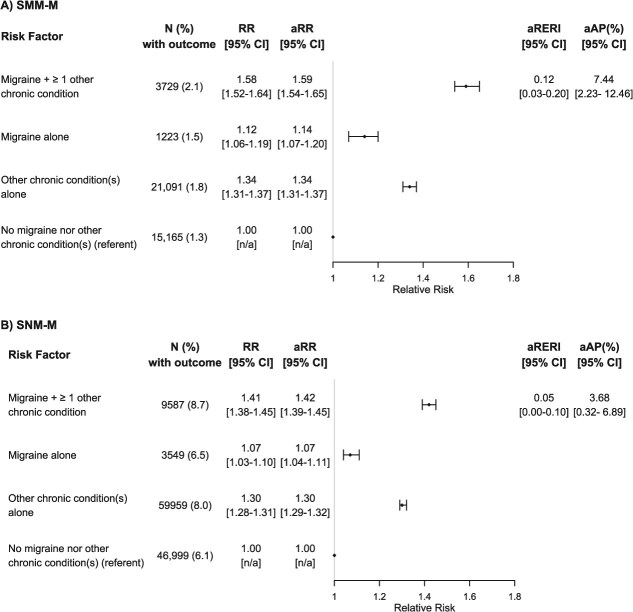
Additive interaction between history of migraine in the five years before conception and any other chronic condition on the risks of (A) SMM-M and (B) SNM-M. Associations adjusted for age, year of conception, parity, neighborhood income quintile, rural residence, immigrant/refugee status, recent history of interpersonal violence, and (for SNM-M only), fetal sex. Abbreviations: AP, attributable proportion due to interaction; RERI, relative excess risk due to interaction; RR, relative risk; SMM-M, severe maternal morbidity or mortality; SNM-M, severe neonatal morbidity or mortality.

The incidence of SMM-M was 1.6% among all pregnancies, while SNM-M affected 7.1% of newborns. Relative risks of SMM-M were highest among individuals with both migraine and other chronic conditions (aRR 1.59, 95% CI 1.54-1.65), followed by other chronic conditions alone (aRR 1.34, 1.31-1.37), and migraine alone (aRR 1.14, 1.07-1.20), compared to neither migraine nor other chronic conditions. Positive additive interaction—or the *excess* risk in the doubly exposed group that is attributable to interaction—explained 7.4% (95% CI 2.2-12.5) of the risk of SMM-M in the group with migraine and other chronic conditions (aRERI 0.12, 95% CI 0.03-0.20). For SNM-M, risks were highest for migraine and other chronic conditions (aRR 1.42, 95% CI 1.39-1.45), followed by other chronic conditions alone (aRR 1.30, 95% CI 1.29-1.32), and migraine alone (aRR 1.07, 95% CI 1.04-1.11) compared to neither migraine nor other chronic conditions. We observed a small positive additive interaction effect (aRERI 0.05, 95% CI 0.01-0.11; aAP 3.7%, 95% CI 0.3-6.9) (Figure 1).

**Figure 2:**
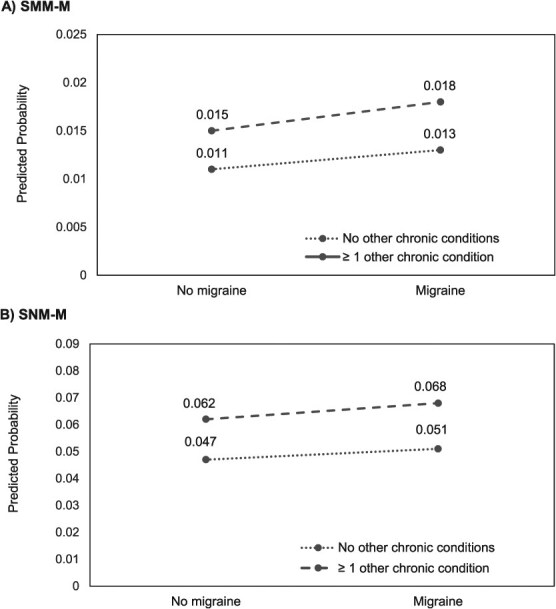
Predicted probability of (A) severe maternal morbidity or mortality (SMM-M) and (B) severe neonatal morbidity or mortality (SNM-M) among individuals with migraine and other chronic condition(s), migraine alone, other chronic conditions alone, and neither migraine nor other chronic conditions, adjusted for confounders (all covariates set at reference values). Probabilities within strata of “other chronic conditions” are joined by lines for the purpose of clearly visualizing additive interactions. Abbreviations: SMM-M, severe maternal morbidity or mortality; SNM-M, severe neonatal morbidity or mortality.

To illustrate the interaction between migraine and other chronic conditions adjusting for confounders, predicted probabilities of SMM-M and SNM-M, setting all covariates equal to their reference values, are shown in Figure 2.

Finally, as illustrated in Table S8, it is probable that the present study underestimated the association between migraine and study outcomes by up to fourfold given potential misclassification of migraine status.

### Additional findings

**Figure 3:**
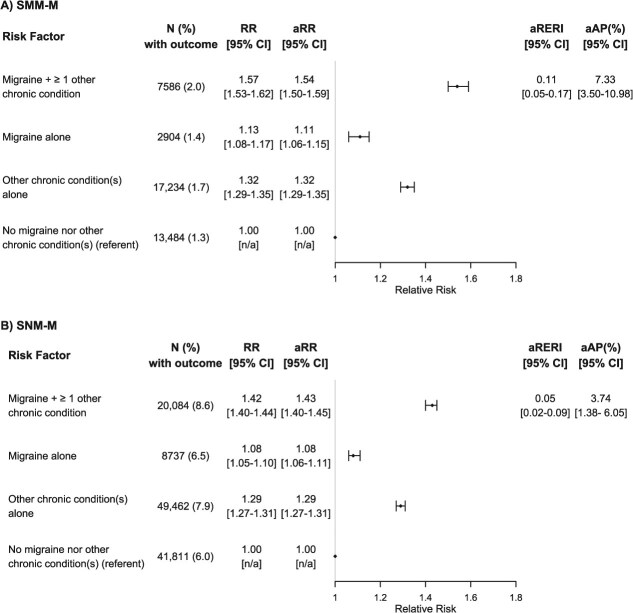
Additive interaction between *lifetime history* of migraine any time before conception and any other chronic condition(s) on the risks of (A) SMM-M and (B) SNM-M. Associations adjusted for age, parity, neighborhood income quintile, rural residence, immigrant/refugee status, recent history of interpersonal violence, and (for SNM-M only), fetal sex. Abbreviations: AP, attributable proportion due to interaction; RERI, relative excess risk due to interaction; RR, relative risk; SMM-M, severe maternal morbidity or mortality; SNM-M, severe neonatal morbidity or mortality.

**Figure 4:**
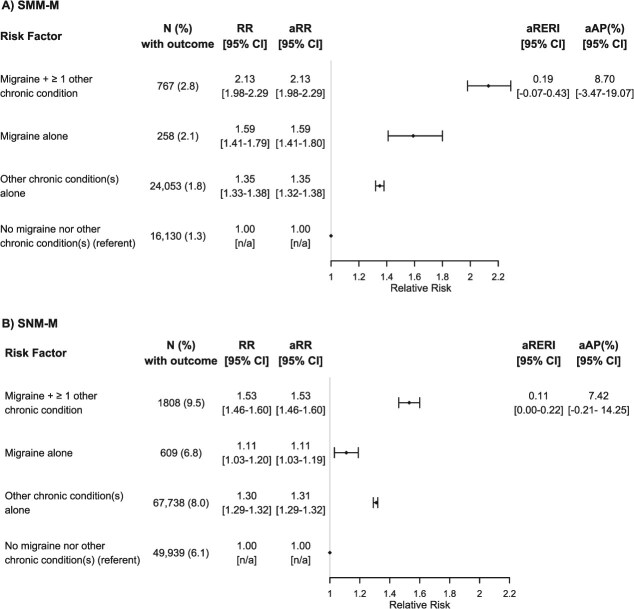
Additive interaction between migraine *during pregnancy* and any other chronic condition(s) on the risks of (A) SMM-M and (B) SNM-M. Associations adjusted for age, parity, neighborhood income quintile, rural residence, immigrant/refugee status, recent history of interpersonal violence, and (for SNM-M only), fetal sex. Abbreviations: AP, attributable proportion due to interaction; RERI, relative excess risk due to interaction; RR, relative risk; SMM-M, severe maternal morbidity or mortality; SNM-M, severe neonatal morbidity or mortality.

Results were similar using a lifetime lookback period to define migraine (Figure 3) and for migraine in pregnancy, except that the aRR of SMM-M among those with migraine alone (aRR 1.59, 95% CI 1.41-1.80) and doubly exposed (aRR 2.13, 95% CI 1.98-2.29) were notably higher than the main analyses when considering migraine during pregnancy (Figure 4).

**Figure 5:**
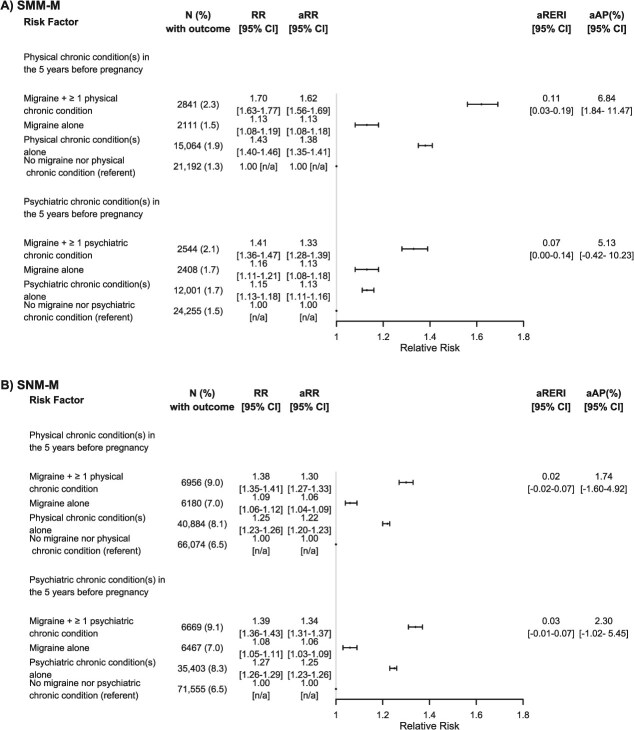
Additive interaction between history of migraine in the 5 years before conception and physical and psychiatric chronic conditions on the risks of (A) SMM-M and (B) SNM-M. Associations adjusted for age, year of conception, parity, neighborhood income quintile, rural residence, immigrant/refugee status, recent history of interpersonal violence, and (for SNM-M only), fetal sex. Abbreviations: AP, attributable proportion due to interaction; RERI, relative excess risk due to interaction; RR, relative risk; SMM-M, severe maternal morbidity or mortality; SNM-M , severe neonatal morbidity or mortality.

When we examined physical and psychiatric chronic conditions separately, patterns were similar to the main analyses. However, we found evidence of positive additive interaction for migraine and any physical chronic condition only on the risk of SMM-M (aRERI 0.11, 95% CI 0.03-0.19; aAP 6.8%, 95% CI 1.8-11.5) (Figure 5).

When we examined chronic conditions by body system, notable findings included the relatively high aRR of SMM-M in those doubly exposed to migraine and cardiovascular (aRR 2.25, 95% CI 2.05-2.47) and cerebrovascular (aRR 1.88, 95% CI 1.52-2.32) disease (Table S9). For SNM-M, aRR were highest for those doubly exposed to migraine and cardiovascular disease (aRR 1.56, 95% CI 1.46-1.67) and sleep disorders (aRR 1.81, 95% CI 1.39-2.36; Table S10).

Notable findings in analyses of individual indicators of SMM-M (Table S11) included the presence of positive additive interaction between migraine and other chronic conditions for maternal ICU admission (aAP 17.9%, 95% CI 7.12-27.0). For SNM-M indicators, the magnitude and direction of results were similar to main analyses (Table S12). Overall, estimates of aRERI and aAP in these analyses had wide confidence intervals, indicating imprecision.

The magnitude of the associations was similar to the main analyses in SMM-M analyses restricted to livebirths and stillbirths to additionally adjust for multiple birth status (Figure S2), but the incidence of SMM-M was slightly higher in each of the four exposure groups.

Results from analyses restricted to individuals born in Ontario to additionally adjust for adverse childhood experiences were similar to main analyses except that there was no association between migraine alone and SMM-M (aRR 0.97, 95% CI 0.81-1.15; Figure S3).

## Discussion

This large population-based study found that risks of SMM-M and SNM-M were greatest among individuals with co-occurring migraine and other chronic conditions, followed by other chronic conditions alone and migraine alone, compared to neither type of condition. There was some evidence of positive additive interaction between migraine and other chronic conditions, although synergistic effects were small. Nevertheless, the elevated risk in those doubly exposed to migraine and other chronic conditions is important since over two-thirds of individuals with migraine had *at least one* other chronic conditions (6.8% of the cohort). Findings were consistent overall for physical and psychiatric chronic conditions, and generally robust in sensitivity analyses that varied the definitions of migraine, the outcome, and the included confounders.

Migraine is an important independent risk factor for maternal and neonatal complications,^4^^,^^5^ but few studies have considered the separate and combined impacts of migraine and comorbidity on perinatal outcomes.^16^^-^^18^ The findings of the present study align with those of prior investigations, which reported the highest risks of hypertensive disorders of pregnancy among individuals with both migraine and comorbid health conditions.^16^^-^^18^ Our findings add to this literature by expanding the range of comorbidities examined and by focusing on SMM-M and SNM-M—rare but serious indicators of adverse maternal and neonatal outcomes.

There are several possible explanations for the observed association between pre-pregnancy migraine, comorbidity, and SMM-M and SNM-M. Physiological differences in vasculature between individuals with and without migraine have been documented, even between migraine attacks.^6^^,^^7^ Such abnormalities are implicated in maternal complications such as preeclampsia^40^ and in cardiovascular and cerebrovascular events^41^^,^^42^ that are leading causes of maternal mortality.^43^ How these abnormalities relate to SNM-M is less clear. However, preterm birth, low birthweight, cerebral infarction, and seizures may also relate to maternal vascular events^44^ and prior research has reported associations between migraine and preterm birth, low birthweight, and seizures.^5^^,^^45^ Multimorbidity literature suggests that the compounded pathophysiological effects of having multiple chronic conditions, treatment complexities and associated stress, as well as the use of multiple prescriptions resulting in adverse drug events can lead to excess risk of adverse health outcomes.^46^ Such factors could explain the greatest risks of SMM-M and SNM-M (and modest additive interaction) observed in individuals with migraine and other chronic conditions, wherein the conditions examined also have known effects on maternal and neonatal health.^47^ Studies vary widely in the breadth of chronic conditions examined, making it difficult to compare the results of the present study to previous research using different lists of chronic conditions. Nevertheless, findings from previous studies and the present study are complementary in that they demonstrate the presence of multiple chronic conditions in pregnancy is important, whether considering multimorbidity broadly or a particular index condition (such as migraine) and its comorbidities.

While our findings underscore the importance of comorbidity among pregnant migraine patients, even migraine present in isolation was associated with increased risk of SMM-M and SNM-M in the overall cohort. While relative risks were lower than those reported for other, rarer neurological diseases such as epilepsy and traumatic brain injury,^48^^,^^49^ migraine is highly prevalent among individuals of childbearing age.^1^ Our findings were robust in most sensitivity analyses that varied the definitions of the exposures, outcomes, and covariates. However, it is notable that the association between migraine alone and SMM-M was attenuated and no longer statistically significant when history of any adverse childhood experience was included as a covariate. It is possible that the association between migraine and SMM-M could be fully explained by a history of adverse childhood experiences in this sub-cohort, although we did not undertake a mediation analysis. However, the crude RR was comparable to the adjusted RR in the sub-cohort, suggesting that attenuation of the association compared to the main analysis was due to differences between the sub-cohort and the overall cohort (not adjustment). The sub-cohort was restricted to individuals born in Ontario who were, by definition, younger and less likely to be immigrants than those in the overall cohort. Future studies should continue to examine how history of adverse childhood experiences impacts associations between migraine, comorbidity, and adverse perinatal outcomes.

### Strengths and limitations

To our knowledge, this study is the first to examine the separate and combined impacts of migraine and other chronic conditions on the risks of SMM-M and SNM-M. We used a population-based cohort representative of >97% of all births in Ontario,^50^ to examine these rare but important outcomes.

While we utilized a migraine algorithm previously validated in the population of interest, sensitivity (26.5%) is modest, even when using a lifetime lookback period (49.1%).^23^ We may therefore have misclassified true migraine cases as not having migraine. This misclassification is likely to be non-differential considering we measured migraine diagnoses documented *before* pregnancy. As described in our sensitivity analyses, it is possible that we underestimated the association between migraine and the study outcomes by up to fourfold, meaning that the reported associations are conservative. Such misclassification could have also impacted our estimates of additive interaction (eg, aRERI, aAP)^51^; however, approaches for quantitative bias analysis in the context of additive interaction with a four-level exposure are not well established in available software, and simple quantitative bias analysis commonly runs into computational challenges, such as negative cell counts. Therefore, we were unable to report bias-adjusted results for our additive interaction analyses.

The observed effects may also be affected by unmeasured confounding, owing to residual confounding by individual-level indicators of socioeconomic status, adverse childhood experiences not captured in healthcare data, and race or ethnicity as a proxy for experiences of racism.

We also did not measure potential mechanisms explaining the observed associations, such as controlled versus uncontrolled migraine/other chronic conditions, conception with assisted reproductive technology, medication use in pregnancy, and health behaviors such as smoking.

This study was underpowered in its examination of additive interaction measures for rarer types of chronic conditions examined separately and very rare indicators of SMM, leading to imprecise estimates for some aRERI and aAP estimates. However, these analyses were intended a priori to be additional analyses and viewed as exploratory or hypothesis-generating in the absence of prior literature on this topic.

### Implications

Our findings highlight several avenues for further research. An investigation into the complexity of comorbidity patterns—eg, considering the number of body systems implicated or the presence of discordant comorbidity—could reveal further insights into possible at-risk groups. Studies that collect information on migraine characteristics (eg, aura, chronicity) could also be beneficial in further characterizing possible interactions with other chronic conditions, since such characteristics are associated with comorbidity and adverse perinatal outcomes.^52^ Possible drug–drug interactions among individuals with migraine and other chronic conditions should be examined as a potential mechanism for the increased risks in this group. Finally, considering socioeconomic, environmental, and behavioral characteristics as explanatory versus biasing factors could help to understand who is at highest risk of experiencing SMM-M and SNM-M, since these factors affect comorbidity burden.^53^

These findings also have important clinical implications. Preconception planning focused on chronic disease management is important,^54^ including in the presence of migraine and co-occurring chronic conditions. Preconception planning could provide an opportunity for review of plans to manage migraine and other chronic conditions before pregnancy to ensure medication benefits outweigh the risks and to consider other options to manage symptoms, where possible, such as lifestyle changes (eg, diet, physical activity, sleep) or non-pharmacological treatments (eg, psychotherapy for psychiatric disorders). Perinatal healthcare supports such as more intensive monitoring leading to early identification of complications and plans for delivery at tertiary care centers in those with migraine and other chronic conditions, given the heightened risks of severe but often avoidable adverse maternal and neonatal outcomes, may also be warranted. Finally, person-centered integrated care approaches that emphasize collaboration between healthcare providers from different specialties may be useful in reducing the risks of SMM-M and SNM-M associated with co-occurring migraine and other chronic conditions. Such models have been applied outside of pregnancy to manage the healthcare needs of patients with multiple chronic conditions^55^ and within pregnancy to provide coordinated maternity care^56^; such collaborative approaches may improve on healthcare systems, which tend to be organized to treat single chronic conditions.^55^

## Conclusion

Individuals with migraine and other chronic conditions have higher risks of SMM-M and SNM-M than those with migraine or other chronic conditions alone. Targeting perinatal healthcare supports toward migraine patients experiencing comorbidity is important to reduce SMM-M and SNM-M risk.

## Supplementary Material

Web_Material_kwag008

## Data Availability

The data are not available for replication via the authors. The dataset from this study is held securely in coded form at ICES. While legal data sharing agreements between ICES and data providers (eg, healthcare organizations and government) prohibit ICES from making the dataset publicly available, access may be granted to those who meet pre-specified criteria for confidential access, available at www.ices.on.ca/DAS (email: das@ices.on.ca). The full dataset creation plan and underlying analytic code are available from the authors upon request, understanding that the computer programs may rely upon coding templates or macros that are unique to ICES and are therefore either inaccessible or may require modification.

## Disclaimer

Parts of this material are based on data and/or information compiled and provided by the Ontario Ministry of Health (MOH), and the Canadian Institute for Health information (CIHI). Parts of this material are based on data and/or information compiled and provided by Immigration Refugees and Citizenship Canada (IRCC) current to September 2020. This document used data adapted from the Statistics Canada Postal CodeOM Conversion File, which is based on data licensed from Canada Post Corporation, and/or data adapted from the Ontario Ministry of Health Postal Code Conversion File, which contains data copied under license from ©Canada Post Corporation and Statistics Canada. Parts of this report are based on Ontario Registrar General (ORG) information on deaths, the original source of which is ServiceOntario. This study is based in part on data provided by Better Outcomes Registry and Network (“BORN”), part of the children’s hospital of Eastern Ontario. The analyses, conclusions, opinions and statements expressed herein are solely those of the authors and do not reflect those of the data sources; no endorsement is intended or should be inferred.

## References

[1] Burch R . Epidemiology and treatment of menstrual migraine and migraine during pregnancy and lactation: a narrative review. *Headache*. 2020;60(1):200-216. 10.1111/head.13665 31579938

[2] Headache Classification Committee of the International Headache Society (IHS) . The International Classification of Headache Disorders, 3rd edition. *Cephalalgia*. 2018;38(1):1-211. 10.1177/0333102417738202 29368949

[3] Steiner TJ, Stovner LJ, Jensen R, et al. Migraine remains second among the world’s causes of disability, and first among young women: findings from GBD2019. *J Headache Pain*. 2020;21(1):137. 10.1186/s10194-020-01208-0 33267788 PMC7708887

[4] Bandoli G, Baer RJ, Gano D, et al. Migraines during pregnancy and the risk of maternal stroke. *JAMA Neurol*. 2020;77(9):1177-1179. 10.1001/jamaneurol.2020.1435 32478828 PMC7265122

[5] Phillips K, Clerkin-Oliver C, Nirantharakumar K, et al. How migraine and its associated treatment impact on pregnancy outcomes: umbrella review with updated systematic review and meta-analysis. *Cephalalgia*. 2024;44(2):03331024241229410. 10.1177/03331024241229410 38317644

[6] Paolucci M, Altamura C, Vernieri F. The role of endothelial dysfunction in the pathophysiology and cerebrovascular effects of migraine: a narrative review. *J Clin Neurol*. 2021;17(2):164-175. 10.3988/jcn.2021.17.2.164 33835736 PMC8053543

[7] Zeller JA, Frahm K, Baron R, et al. Platelet-leukocyte interaction and platelet activation in migraine: a link to ischemic stroke? *J Neurol Neurosurg Psychiatry*. 2004;75(7):984-987. 10.1136/jnnp.2003.019638 15201354 PMC1739108

[8] Thuraiaiyah J, Erritzøe-Jervild M, Al-Khazali HM, et al. The role of cytokines in migraine: a systematic review. *Cephalalgia*. 2022;42(14):1565-1588. 10.1177/03331024221118924 35962530

[9] Rebordosa C, Zelop CM, Kogevinas M, et al. Use of acetaminophen during pregnancy and risk of preeclampsia, hypertensive and vascular disorders: a birth cohort study. *J Matern Fetal Neonatal Med*. 2010;23(5):371-378. 10.3109/14767050903334877 19929241

[10] Chen X, Yang Y, Chen L, et al. Pregnancy outcomes and birth defects in offspring following non-steroidal anti-inflammatory drugs exposure during pregnancy: a systematic review and meta-analysis. *Reprod Toxicol*. 2024;125:108561. 10.1016/j.reprotox.2024.108561 38423229

[11] Ross LE, Grigoriadis S, Mamisashvili L, et al. Selected pregnancy and delivery outcomes after exposure to antidepressant medication: a systematic review and meta-analysis. *JAMA Psychiatry*. 2013;70(4):436-443. 10.1001/jamapsychiatry.2013.684 23446732

[12] Sanghavi M, Rutherford JD. Cardiovascular physiology of pregnancy. *Circulation*. 2014;130(12):1003-1008. 10.1161/CIRCULATIONAHA.114.009029 25223771

[13] Feinstein A . The pre-therapeutic classification of co-morbidity in chronic disease. *J Chronic Dis*. 1970;23(7):455-468. 10.1016/0021-9681(70)90054-8 26309916

[14] Altamura C, Corbelli I, De Tommaso M, et al. Pathophysiological bases of comorbidity in migraine. *Front Hum Neurosci*. 2021;15:640574. 10.3389/fnhum.2021.640574 33958992 PMC8093831

[15] Minen MT, Begasse De Dhaem O, Kroon Van Diest A, et al. Migraine and its psychiatric comorbidities. *J Neurol Neurosurg Psychiatry*. 2016;87(7):741-749. 10.1136/jnnp-2015-312233 26733600

[16] Adeney KL, Williams MA, Miller RS, et al. Risk of preeclampsia in relation to maternal history of migraine headaches. *J Matern Fetal Neonatal Med*. 2005;18(3):167-172. 10.1080/14767050500260566 16272039

[17] Cripe SM, Frederick IO, Qiu C, et al. Risk of preterm delivery and hypertensive disorders of pregnancy in relation to maternal co-morbid mood and migraine disorders during pregnancy. *Paediatr Perinat Epidemiol*. 2011;25(2):116-123. 10.1111/j.1365-3016.2010.01182.x 21281324 PMC3756187

[18] Czerwinski S, Gollero J, Qiu C, et al. Migraine-asthma comorbidity and risk of hypertensive disorders of pregnancy. *J Pregnancy*. 2012;2012:858097. 10.1155/2012/858097 22934185 PMC3425816

[19] Benchimol EI, Smeeth L, Guttmann A, et al. The REporting of studies Conducted using Observational Routinely-collected health Data (RECORD) statement. *PLoS Med*. 2015;12(10):e1001885. 10.1371/journal.pmed.1001885 26440803 PMC4595218

[20] Schull MJ, Azimaee M, Marra M, et al. ICES: data, discovery, better health. *IJPDS*. 2020;4(2):1135. 10.23889/ijpds.v4i2.1135 32935037 PMC7477779

[21] Juurlink D, Preyra C, Croxford R, et al. Canadian Institute for Health Information Discharge Abstract Database: a validation study. ICES. Toronto; 2006. www.ices.on.ca

[22] Joseph KS, Fahey J, Canadian Perinatal Surveillance System. Validation of perinatal data in the Discharge Abstract Database of the Canadian Institute for Health Information. *Chronic Dis Can*. 2009;29(3):96-100. 19527567

[23] Albanese CM, Bondy SJ, Lay C, et al. Use of health administrative data to identify migraine in individuals with a recognized pregnancy: a validation study in Ontario. *Can Epidemiol*. 2025;36(5):599-605. 10.1097/EDE.0000000000001890 40488350

[24] Government of Canada . Canadian Perinatal Surveillance System. canada.ca. Accessed March 4, 2025. https://www.canada.ca/en/public-health/services/injury-prevention/health-surveillance-epidemiology-division/maternal-infant-health/canadian-perinatal-surveillance-system.html

[25] Ray JG, Park AL, Dzakpasu S, et al. Prevalence of severe maternal morbidity and factors associated with maternal mortality in Ontario, Canada. *JAMA Netw Open*. 2018;1(7):e184571. 10.1001/jamanetworkopen.2018.4571 30646359 PMC6324398

[26] Nelson CRM, Ray JG, Auger N, et al. Neonatal adverse outcomes among hospital livebirths in Canada: a national retrospective study. *Neonatology*. 2025;122(1):114-121. 10.1159/000540559 39173602 PMC11809516

[27] Knight HE, Oddie SJ, Harron KL, et al. Establishing a composite neonatal adverse outcome indicator using English hospital administrative data. *Arch Dis Child Fetal Neonatal Ed*. 2019;104(5):F502-F509. 10.1136/archdischild-2018-315147 30487299 PMC6703994

[28] Todd S, Bowen J, Ibiebele I, et al. A composite neonatal adverse outcome indicator using population-based data: an update. *Int J Popul Data Sci*. 2020;5(1):1337 Published 2020 Aug 12. 10.23889/ijpds.v5i1.1337 33644407 PMC7893849

[29] Lebreton E, Menguy C, Fresson J, et al. Measuring severe neonatal morbidity using hospital discharge data in France. *Paediatr Perinat Epidemiol*. 2022;36(2):190-201. 10.1111/ppe.12816 34797588

[30] Misra DP, Guyer B, Allston A. Integrated perinatal health framework. A multiple determinants model with a life span approach. *Am J Prev Med*. 2003;25(1):65-75. 10.1016/s0749-3797(03)00090-4 12818312

[31] Austin PC . Using the standardized difference to compare the prevalence of a binary variable between two groups in observational research. *Commun Stat Simul Comput*. 2009;38(6):1228-1234. 10.1080/03610910902859574

[32] Zou GY, Donner A. Extension of the modified Poisson regression model to prospective studies with correlated binary data. *Stat Methods Med Res*. 2013;22(6):661-670. 10.1177/0962280211427759 22072596

[33] VanderWeele TJ, Lash TL, Rothman KJ. Chapter 26: Analysis of Interaction. In: Rothman KJ, Greenland S, Lash TL, eds. *Modern Epidemiology*. 4th ed. Wolters Kluwer; 2021:619-651.

[34] Zou GY . On the estimation of additive interaction by use of the four-by-two table and beyond. *Am J Epidemiol*. 2008;168(2):212-224. 10.1093/aje/kwn104 18511428

[35] Fox MP, Fink A, Lash TL. Quantitative Bias Analysis Spreadsheet [Microsoft Excel Worksheet]. 2007. Accessed November 15, 2025. https://sites.google.com/site/biasanalysis

[36] SAS Enterprise Guide Version 8.3. SAS Institute Inc; 2020.

[37] R: A Language and Environment for Statistical Computing, Version 4.2.0. R Core Team; 2022.

[38] Mamun A, Biswas T, Scott J, et al. Adverse childhood experiences, the risk of pregnancy complications and adverse pregnancy outcomes: a systematic review and meta-analysis. *BMJ Open*. 2023;13(8):e063826. 10.1136/bmjopen-2022-063826 PMC10401231 37536966

[39] Sikorski C, Mavromanoli AC, Manji K, et al. Adverse childhood experiences and primary headache disorders: a systematic review, meta-analysis, and application of a biological theory. *Neurology*. 2023;101(21):e2151-e2161. 10.1212/WNL.0000000000207910 37879940 PMC10663032

[40] Jakobsen C, Larsen JB, Fuglsang J, et al. Platelet function in preeclampsia: a systematic review and meta-analysis. *Platelets*. 2019;30(5):549-562. 10.1080/09537104.2019.1595561 30983478

[41] Bigal ME, Kurth T, Santanello N, et al. Migraine and cardiovascular disease: a population-based study. *Neurology*. 2010;74(8):628-635. 10.1212/WNL.0b013e3181d0cc8b 20147658 PMC3462501

[42] Adelborg K, Szépligeti SK, Holland-Bill L, et al. Migraine and risk of cardiovascular diseases: Danish population based matched cohort study. *BMJ.* 2018;360:k96. 10.1136/bmj.k96 29386181 PMC5791041

[43] Sprague AE, Roberts NF, Lavin Venegas C, et al. Mortality following childbirth in Ontario: a 20-year analysis of temporal trends and causes. *J Obstet Gynaecol Can*. 2024;46(12):102689. 10.1016/j.jogc.2024.102689 39401573

[44] Owens A, Yang J, Nie L, et al. Neonatal and maternal outcomes in pregnant women with cardiac disease. *JAHA*. 2018;7(21):e009395. 10.1161/JAHA.118.009395 30571384 PMC6404206

[45] Skajaa N, Szépligeti SK, Xue F, et al. Pregnancy, birth, neonatal, and postnatal neurological outcomes after pregnancy with migraine. *Headache*. 2019;59(6):869-879. 10.1111/head.13536 31069791

[46] Vogeli C, Shields AE, Lee TA, et al. Multiple chronic conditions: prevalence, health consequences, and implications for quality, care management, and costs. *J Gen Intern Med*. 2007;22 Suppl 3(S3):391-395. 10.1007/s11606-007-0322-1 18026807 PMC2150598

[47] Rosman AN, van der Vliet-Torij HWH, Hilberink SR. Trends in perinatal outcomes of women with chronic medical conditions: a 10-year population-based study in the Netherlands. *Midwifery*. 2023;118:103572. 10.1016/j.midw.2022.103572 36587471

[48] Panelli DM, Leonard SA, Kan P, et al. Association of epilepsy and severe maternal morbidity. *Obstet Gyneco*. 2021;138(5):747-754. 10.1097/AOG.0000000000004562 PMC8542621 34619720

[49] Adams RS, Akobirshoev I, Brenner LA, et al. Pregnancy, fetal, and neonatal outcomes among women with traumatic brain injury. *J Head Trauma Rehabil*. 2023;38(3):E167-E176. 10.1097/HTR.0000000000000807 36731040 PMC10102258

[50] Statistics Canada . Table 13-10-0429-01 live births and fetal deaths (stillbirths), by place of birth (hospital or non-hospital). Published online. 2021. 10.25318/1310042901-eng

[51] Jurek AM, Greenland S, Maldonado G, et al. Proper interpretation of non-differential misclassification effects: expectations vs observations. *Int J Epidemiol*. 2005;34(3):680-687. 10.1093/ije/dyi060 15802377

[52] Blumenfeld A, Varon S, Wilcox T, et al. Disability, HRQoL and resource use among chronic and episodic migraineurs: results from the International Burden of Migraine Study (IBMS). *Cephalalgia*. 2011;31(3):301-315. 10.1177/0333102410381145 20813784

[53] Valderas JM, Starfield B, Sibbald B, et al. Defining comorbidity: implications for understanding health and health services. *Ann Fam Med*. 2009;7(4):357-363. 10.1370/afm.983 19597174 PMC2713155

[54] Cragan JD, Friedman JM, Holmes LB, et al. Ensuring the safe and effective use of medications during pregnancy: planning and prevention through preconception care. *Matern Child Health J*. 2006;10(S1):S129-S135. 10.1007/s10995-006-0102-2 16850277 PMC1592140

[55] Van der Heide I, Snoeijs S, Melchiorre M, et al. Innovating Care for People with Multiple Chronic Conditions in Europe: An Overview. Institute for Health Services Research, 2015. https://www.nivel.nl/en/publicatie/innovating-care-people-multiple-chronic-conditions-europe-overview. Accessed June 19, 2025.

[56] Kroll-Desrosiers AR, Crawford SL, Moore Simas TA, et al. Improving pregnancy outcomes through maternity care coordination: a systematic review. *Womens Health Issues*. 2016;26(1):87-99. 10.1016/j.whi.2015.10.003 26586143

