# Supplementary material for: Migraine, comorbidity, and risks of severe maternal and neonatal morbidity or mortality: a population-based cohort study
Source: Am J Epidemiol. 2026 Jan 15;195(7):1933–43. doi: 10.1093/aje/kwag008 (PMC13343373; doi:10.1093/aje/kwag008)
Supplement: Web_Material_kwag008 [file web_material_kwag008.docx]

**SUPPLEMENTAL MATERIAL**

**Title:** Migraine, comorbidity, and risks of severe maternal and neonatal morbidity or mortality: A population-based cohort study

**Authors:** Carmela Melina Albanese, Susan J Bondy, Christine Lay, Manav V. Vyas, Zhiyin Li, Jun Guan, Hilary K. Brown

**Included Materials:**

**Supplemental Tables**

[**Table S1.** Description of ICES databases 2](#_Toc219122943)

[**Table S2.** Identification of documented pregnancies, Ontario, Canada, 2007-2022 4](#_Toc219122944)

[**Table S3.** Characteristics of algorithms used to identify migraine 5](#_Toc219122945)

[**Table S4.** Measurement of chronic conditions in the 5 years before conception (unless otherwise specified in algorithm). 6](#_Toc219122946)

[**Table S5.** Measurement of outcome variables 14](#_Toc219122947)

[**Table S6.** Measurement of covariates 20](#_Toc219122948)

[**Table S7.** Comparison of characteristics among pregnancies to individuals with migraine and ≥ 1 chronic condition, migraine alone, and other chronic condition(s) alone, compared with pregnancies to individuals with neither migraine nor other chronic conditions, Ontario, Canada, 2007-2022. 21](#_Toc219122949)

[**Table S8.** Estimates of RR among “migraine alone” group adjusted for bias due to migraine misclassification. 23](#_Toc219122950)

[**Table S9.** Additive interaction between migraine in the 5 years before conception and type of chronic condition (grouped by primary body system affected) on the risk of SMM-M 24](#_Toc219122951)

[**Table S10.** Additive interaction between migraine in the 5 years before conception and type of chronic conditions (grouped by primary body system affected) on the risk of SNM-M. 28](#_Toc219122952)

[**Table S11.** Additive interaction between migraine in the 5 years before conception and any other chronic conditions on the risk of individual indicators of SMM. 32](#_Toc219122953)

[**Table S12.** Additive interaction between migraine in the 5 years before conception and any other chronic condition on the risks of individual indicators of SNM. 37](#_Toc219122954)

**Supplemental Figures**

[**Figure S1.** Conceptual framework of migraine, associated chronic diseases, and maternal and neonatal outcomes. 45](#_Toc219122381)

[**Figure S2.** Additive interaction between migraine in the 5 years before conception and any other chronic condition on the risk of SMM-M among N=1,734,729 pregnancies ending in a livebirth or stillbirth and additionally adjusted for multiple birth status. 46](#_Toc219122382)

[**Figure S3.** Additive interaction between migraine in the 5 years before conception and any other chronic condition on the risks of A) SMM-M and B) SNM-M among individuals born in Ontario and additionally adjusted for history of adverse childhood experiences. 48](#_Toc219122383)

**References**

**References for supplemental material**…………………………………………………………………..49

**Table S1.** Description of ICES databases

| **Database** | **Description and data** | **Inception** |
| --- | --- | --- |
| Assistive Devices Program (ADP) | Information on support and funding to Ontario residents with long-term physical disabilities. | 2000 |
| Better Outcomes Registry Network (BORN) | Ontario’s pregnancy, birth and childhood registry and network. Collects data about births in the province, including prenatal health behaviours. | 2006 |
| Canadian Institute for Health Information Discharge Abstract Database (CIHI-DAD) | Hospital admissions and discharge data. Diagnostic and procedure data coded using:   - ICD-9 and CCP codes (1988-2001) - ICD-10-CA and CCI codes (2022-Present) | 1988 |
| Immigration, Refugees, and Citizenship Canada (IRCC) Permanent Residents database | Data about immigration records | 1985 |
| ICES-derived cohorts | Population-based cohorts of chronic disease based on validated algorithms developed at ICES. Those used in this study include: Asthma Dataset (ASTHMA), Chronic Obstructive Pulmonary Disease (COPD) database, Congestive Heart Failure (CHF) database, Ontario Crohn’s and Colitis Dataset (OCCC), Ontario Diabetes Dataset (ODD), Ontario Hypertension Dataset (HYPER), Ontario Myocardial Infarction Dataset (OMID), and the Ontario Rheumatoid Arthritis Dataset (ORAD). | Variable; from 1991-1993 |
| ICES Physician Database (IPDB) | Information about physicians practicing in Ontario | 1992 |
| MOMBABY database | Linked maternal and newborn records for all hospital births (>97% of births in Ontario) (2) | 1988 |
| National Ambulatory Care Reporting System (NACRS) | Emergency department visits; Diagnostic and procedure data coded using:   - ICD-9 and CCP codes (1988-2001) - ICD-10-CA and CCI codes (2022-Present) | 2002 |
| Ontario Health Insurance Plan Claims Database (OHIP) | Outpatient physician visits; Diagnostic data coded using 3-digit physician billing codes, based on ICD-9 | 1991 |
| Ontario Mental Health Reporting System (OMHRS) | Receipt of inpatient mental heath services in Ontario; Diagnostic data coded using DSM | 2005 |
| Ontario Registrar General Database (ORGD) | Vital statistics on death | 1990 |
| Registered Persons Database (RPDB) | Date of birth and residential postal code | 1991 |
| Same Day Surgery database (SDS) | Information on administrative, diagnostic and procedure patient-level information for all day surgery and outpatient clinic visits | 1991 |
| Statistics Canada Census data (CENSUS) | Ontario Census Area Profiles; variables defined according to residential postal code | 1991 |

Abbreviations: CCI = Canadian Classification of Health Interventions; CCP = Canadian Classification of Diagnostic, Therapeutic, and Surgical Procedures; DSM= Diagnostic and Statistical Manual of Mental Disorders; ICD = International Classification of Diseases and Related Health Problems; IRCC = Immigration, Refugees and Citizenship Canada.

**Table S2.** Identification of documented pregnancies, Ontario, Canada, 2007-2022

| **Pregnancy outcome** | **Codes** | **Data sources** |
| --- | --- | --- |
| Livebirth | MOMBABY Stillbirth=”F” | MOMBABY |
| Stillbirth | MOMBABY Stillbirth=”T” | MOMBABY |
| Induced abortion* | ICD-9: 635, 636; ICD-10: O04 or O08 and CCP: 81.01, 87.0-87.1, 87.21, or 87.29; CCI: 5CA20FK, 5CA24, 5CA88, or 5CA89 (INATSTAT not equal to A); OHIP: 635 or 895 (feecode with feesuff = A or B) and S752, S785, A920, or P001 | CIHI-DAD, CIHI-SDS, NACRS, OHIP |
| Miscarriage* | ICD-9: 632, 633, 634; ICD-10: O00, O02.1, or O03, O36.4; OHIP: 632, 633, 634, or 640 and A920 or P001; A922; 632, 633, 634, or 640 and S752 or S785; or S756, S768, S770, or S784 | CIHI-DAD, CIHI-SDS, NACRS OHIP |
| Threatened abortion^a^ | ICD-9: 640; ICD-10: O20; OHIP: 640 | CIHI-DAD, NACRS, OHIP |

Abbreviations: CCI = Canadian Classification of Health Interventions; CIHI-DAD = Canadian Institute for Health Information Discharge Abstract Database; ICD = International Classification of Diseases and Related Health Problems; OHIP = Ontario Health Insurance Plan

a Threatened abortion, induced abortion, or miscarriage within 90 days of another pregnancy outcome were not counted, as this could be a misclassified hemorrhage related to the prior pregnancy. Suspected cases of abortion and miscarriages were excluded. Only threatened abortion without evidence of the pregnancy ending in livebirth, stillbirth, induced abortion or miscarriage were counted; this variable is intended to capture people with evidence of a miscarriage who might have had that miscarriage at home or somewhere else outside of the health care system (3).

**Table S3.** Characteristics of algorithms used to identify migraine

| **Algorithm details** | **Sensitivity (%), 95%CI** | **Specificity (%), 95%CI** | **PPV (%), 95%CI** | **NPV (%), 95%CI** | **Percent agreement (%)** |
| --- | --- | --- | --- | --- | --- |
| ≥ 1 physician visit (OHIP: 346), emergency department visit, or hospital admission (ICD-10: G43) with migraine diagnosis code *in the 5 years before conception* | 26.5 [23.3-29.7] | 93.5 [92.5-94.5] | 46.7 [41.2-52.3] | 85.6 [84.3-86.9] | 81.7 |
| ≥ 1 physician visit (OHIP: 346), emergency department visit, or hospital admission (ICD-10: G43) with migraine diagnosis code *ever* (from birth or entry into OHIP) until estimated conception date | 49.1 [45.4-52.8] | 84.8 [83.5-86.2] | 41.0 [37.3-44.6] | 88.6 [87.4-90.0] | 78.5 |

Abbreviations: PPV = positive predictive value, NPV = negative predictive value

Sensitivity, specificity, PPV, NPV, and percent agreement were assessed based on comparison to self-reported migraine diagnosis (4)

**Table S4.** Measurement of chronic conditions in the 5 years before conception (unless otherwise specified in algorithm).

| **Variable** | **Codes** | **Data source(s)** | **Body system** | **Prevalence in cohort** |
| --- | --- | --- | --- | --- |
| Asthma (5) | 2 physician (OHIP: 493) or emergency department visits or 1 hospital admission (ICD-9: 493; ICD-10: J45, J46) in 2 years before conception | ASTHMA registry, CIHI-DAD, NACRS, OHIP | Respiratory | 7.0% |
| Atrial fibrillation (6) | 4 physician visits separated by 30 days (OHIP: 427) or 1 hospital admission or ED visit (ICD-9: 427.3; ICD-10: I48) or procedure billing code for cardioversion (Z437) | CIHI-DAD, NACRS, OHIP | Cardiovascular | 0.2% |
| Attention deficit/ hyperactivity disorder (7) | 2 physician visits (OHIP: 314) or emergency department visits or 1 hospital admission (ICD-9: 314; ICD-10: F90) ever since database inception | CIHI-DAD, NACRS, OHIP | Neurodevelopmental^a^ | 1.9% |
| Autism (8) | 2 physician visits (OHIP: 299) or 1 hospitalization or ED visit (ICD-9: 299; ICD-10: F84.0, F84.1, F84.3, F84.4, F84.5, F84.8, F84.9) ever since database inception | CIHI-DAD, NACRS, OHIP | Neurodevelopmental^a^ | 0.1% |
| Chronic hypertension (9) | One hospitalization (ICD-9: 401-405; ICD-10: I10-I13, I15) or one physician visit (OHIP: 401-405) followed by an additional physician visit or hospitalization within 2 years (excluding gestational hypertension) | HYPER registry, CIHI-DAD, OHIP | Cardiovascular | 2.0% |
| Chronic liver disease (10) | 2 physician visits (OHIP: 571) or 1 hospitalization or ED visit (ICD-9: 571; ICD-10: K70.0, K70.2, K70.3, K71.7, K73, K74.6, K75.4, K75.8, K75.9, K76.0, B18) | CIHI-DAD, NACRS, OHIP | Metabolic | 0.2% |
| Chronic obstructive pulmonary disease (11) | 1 physician visit (OHIP: 491, 492, 496) or ED visit or hospitalization (ICD-9: 491, 492, 496; ICD-10: J41, J43, J44) | COPD registry, CIHI-DAD, NACRS, OHIP | Respiratory | 0.2% |
| Congestive heart failure (12) | 1 physician visit (OHIP: 428 or feecode Q050) or emergency visit or hospitalization (ICD-9: 428.0, 428.1, 428.9; ICD-10: I50.0, I50.1, I50.9) followed by a second record from either source in 1 year | CHF registry; CIHI-DAD, NACRS, OHIP | Cardiovascular | 0.0% |
| Coronary syndrome (13) | 2 physician visits (OHIP: 412, 413) or 1 emergency department visit or hospitalization (ICD-9: 412, 413; ICD-10: I20-I25) | CIHI-DAD, NACRS, OHIP | Cardiovascular | 0.3% |
| Diabetes (type 1 or type 2) (14) | If < 19 years: 4 physician visits (OHIP: 250 or feecode Q040, K029, K030, K045, K046) within 2 years and at least 1 of these before 19^th^ birthday; if ≥ 19 years: 2 physician visits (OHIP: 250) or 1 procedure (Q040, K029, K030, K045, K046) or hospital admission (ICD-9: 250; ICD-10: E10, E11, E13, E14) (excluding gestational diabetes) | ODD registry, CIHI-DAD, NACRS, OHIP | Metabolic | 1.5% |
| Endometriosis (15) | 2 physician visits (OHIP: 617) or 1 emergency department or hospitalization (ICD-9: 617; ICD-10: N80), or a surgical procedure coded as a diagnosis of endometriosis (ICD-9: 617; ICD-10: N80) | CIHI-DAD, NACRS, OHIP | Non-migraine chronic pain | 1.0% |
| Epilepsy (16) | 3 physician visits separated by at least 30 days within a 2-year period (OHIP: 345) or 1 ED visit or hospital admission (ICD-9: 345.x except 345.2 and 345.3; ICD-10: G40) | CIHI-DAD, NACRS, OHIP | Neurologic | 0.4% |
| Fibromyalgia (17) | 2 physician visits (OHIP: 729) or 1 ED visit or hospitalization (ICD-9: 729; ICD-10: M79.7), *per Redelmeier et al J Rheumatol 2015; 42(8): 1502-1510 [not a validation study, but lists codes]* | CIHI-DAD, NACRS, OHIP | Non-migraine chronic pain | 0.6% |
| Inflammatory bowel disease (18) | 5 physician visits (OHIP: 555, 556) or ED visits or hospitalizations (ICD-9: 555, 556; ICD-10: K50, K51) in 4 years | OCCC registry, CIHI-DAD, NACRS, OHIP | Autoimmune | 0.5% |
| Irritable bowel syndrome (19) | 2 physician visits (OHIP: 564) at least 6 months apart or 1 ED visit or hospitalization (ICD-9: 564.1; ICD-10: K58) | CIHI-DAD, NACRS, OHIP | Gastrointestinal | 1.2% |
| Low back pain (20) | 2 physician visits (OHIP: 724) or 1 spinal imaging procedure (feecode X025, X202, X203, X027, X204, X028, X205, X206, X032, X033, X031, X034, X207, X035, X208 or CCI 3SC10KM, 3SC10VA, 3SC10VN, 3SE10VK, 3SC12AY, 3SC12VA, 3SE12VA, 3SE12VK, 3SF12VA, 3SF12VL, 3SF10VA, 3SF10VL) or 1 ED visit or hospitalization (ICD-9: 721.3, 722.10, 722.52, 722.93, 722.73, 722.83, 724.02, 724.3, 724.2, 724.5, 724.6, 724.70, 724.71, 724.79, 724.8, 724.9, 739.3, 739.4, 846.0, 846.1, 846.2, 846.3, 846.8, 846.9, 847.2, 847.3, 847.4, 847.9; ICD-10: M47.26, M47.27, M47.28, M47.86, M47.87, M47.88, M47.96, M47.97, M47.98, M48.06, M48.07, M48.08, M48.86, M48.87, M48.88, M48.96, M48.97, M48.98, M51.0, M51.1, M51.2, M51.3, M51.9, M53.3, M53.86, M53.87, M53.88, M54.10, M54.16, M54.17, M54.18, M54.19, M54.3, M54.4, M54.5, M54.8, M54.9, M99.83, M99.93, M99.03, M99.84, M99.94, M99.04, S33.5, S33.6, S33.7, S33.8), *per Wong et al. Pain 2021; 219-226.* | CIHI-DAD, NACRS, OHIP | Non-migraine chronic pain | 9.6% |
| Mood or anxiety disorders (21) | ≥2 physician visits or ≥1 ED visits or hospitalizations:  a. Psychiatrist [SPEC=19] and outpatient (LOCATION: O, L, H, P) and non-lab service [substr(FEECODE,1,1) ne 'G'] OR Family physician / general practitioner [SPEC=00] /pediatrician [SPEC=26] and mental illness or addiction diagnosis code [DXCODE] and outpatient (LOCATION: O, L, H, P) and non-lab service [substr(FEECODE,1,1) ne 'G'] OR pediatrician [SPEC=26] and undefined location (LOCATION=U) and MHA diagnostic code [DXCODE] and fee code (FEECODE=K122 or K123 or K704), where DXCODES = 296, 300, 309, 311  b. CIHI-DAD/NACRS: ICD-10: Before 2016/17: DX10CODE1 F30-F34, F38-F43, F48.8, F48.9, F53.0, F93.1-F93.2; ICD-9: 296.x (all 296 codes), 300, 300.0x, 300.2x, 300.3x, 300.4x, 301.13, 308.3x, 309.0x, 309.24, 309.28, 309.3x, 309.4x, 309.8x, 309.9x, 311; 2016/17-present: DX10CODE1 F06.3, F06.4, F30-F34, F38-F43, F42.x, F45.2, F63.3, F53.0, F93.0-F93.2, F94.0-F94.2; ICD-9: 296 (all 296 codes), 300, 301.13, 311.x, 308.3x, 309.0x, 309.24, 309.28, 309.3x, 309.4x, 309.8x, 309.9x, 311.x  c. OMHRS: Before 2016/17: AXIS1_DSM4CODE_DISCH1 = 296.x (all 296 codes), 300, 300.0x, 300.2x, 300.3x, 300.4x, 301.13, 308.3x, 309.0x, 309.24, 309.28, 309.3x, 309.4x, 309.8x, 309.9x, 311; Provisional: 6, 7, 15; 2016/17-2018/19: DSM5CODE_DISCH1 = 293.83, 293.84, 296.x (all 296 codes), 300, 300.0x, 300.2x, 300.3x, 300.4x, 300.7x, 301.13, 308.3x, 309, 309.0x, 309.21, 309.24, 309.28, 309.3x, 309.4x, 309.81, 309.89, 309.9x, 311.x, 312.39, 313.23, 313.89, 625.4, 698.4x, Provisional = 3-7; 2019/20 to present: ICD10CMCODE_DISCH1=F06.3, F06.4, F06.8, F31-F34, F40.0-F40.2, F41.0, F41.1, F41.8, F41.9, F42.2-F42.4, F42.8, F42.9, F43.0-F43.2, F43.8, F43.9, F45.2, F63.3, F94.1, F94.2, F93.0, F94.0-F94.2; Provisional = 3-7 | CIHI-DAD, NACRS,  OHIP, OMHRS | Psychiatric | 28.4% |
| Multiple sclerosis (22) | 5 physician visits (OHIP: 340) or 1 hospital admission or ED visit (ICD-9: 340, ICD-10: G35) | CIHI-DAD, NACRS, OHIP | Autoimmune | 0.2% |
| Myocardial infarction (23) | 1 hospitalization (ICD-9: 410; ICD-10: I21) | CIHI-DAD, OMID registry | Cardiovascular | 0.0% |
| Obesity (24,25) | If linked to BORN: BMI – NIDAY period = BMI: Obese ≥ 30.0, Other < 30.0; BMI – BIS period: MATERNAL_BMI = Obese ≥ 30.0; Other < 30.0. Where BMI/MATERNAL_BMI is missing: 2 physician visits (OHIP: 278) or 1 emergency department visit or hospital admission (ICD-9: 278; ICD-10: E66) | CIHI-DAD, NACRS, OHIP | Metabolic | 11.0% |
| Obstructive sleep apnea (26) | ICES concept for “Obstructive sleep apnea” case identification | CIHI-DAD, NACRS, OHIP, SDS, IPDB, ADP | Sleep disorder | 0.1% |
| Osteoarthritis (27) | 2 physician visits (OHIP: 715) or 1 ED visit or hospitalization (ICD-9: 715; ICD-10: M15-M19) | CIHI-DAD, NACRS, OHIP | Non-migraine chronic pain | 0.8% |
| Other mental illness (21) | ≥2 physician visits or ≥1 ED visits or hospitalizations:  a. Psychiatrist [SPEC=19] and outpatient (LOCATION: O, L, H, P) and non-lab service [substr(FEECODE,1,1) ne 'G'] OR Family physician / general practitioner [SPEC=00] /pediatrician [SPEC=26] and mental illness or addiction diagnosis code [DXCODE] and outpatient (LOCATION: O, L, H, P) and non-lab service [substr(FEECODE,1,1) ne 'G'] OR pediatrician [SPEC=26] and undefined location (LOCATION=U) and MHA diagnostic code [DXCODE] and fee code (FEECODE=K122 or K123 or K704), where DXCODES = 301, 302, 306, 307, 309, 313-315  b. CIHI-DAD/NACRS: ICD-10: DX10CODE1 All other F06-F99 not included in other categories of mental illness, and excluding IDD diagnoses (F70-73, F78, F79, F84.0, F84.1, F84.3-F84.9) and sleep disorders (F51); ICD-9: DXCODE1 All other 293-302 or 306-319 not included in other categories of mental illness, and excluding IDD diagnoses (299, 317-319) and sleep disorders (307.4, 307.41, 307.42, 307.45, 307.46, 307.47, 307.48, 307.49)  c. OMHRS: All other OMHRS not included in other categories of mental illness, and excluding IDD diagnoses (299, 317-319) and sleep disorders (307.4, 307.41, 307.42, 307.45, 307.46, 307.47, 307.48, 307.49) | CIHI-DAD, NACRS, OHIP, OMHRS | Psychiatric | 5.1% |
| Psoriasis (28) | 1 physician visit (OHIP: 696) or 1 ED visit or hospitalizastion (ICD-9: 696.0, 696.1; ICD-10: L40) | CIHI-DAD, NACRS, OHIP | Autoimmune | 1.4% |
| Psychotic mental illness (21) | Please use ≥2 physician visits or ≥1 ED visits or hospitalizations.  a. Psychiatrist [SPEC=19] and outpatient (LOCATION: O, L, H, P) and non-lab service [substr(FEECODE,1,1) ne 'G'] OR Family physician / general practitioner [SPEC=00] /pediatrician [SPEC=26] and mental illness or addiction diagnosis code [DXCODE] and outpatient (LOCATION: O, L, H, P) and non-lab service [substr(FEECODE,1,1) ne 'G'] OR pediatrician [SPEC=26] and undefined location (LOCATION=U) and MHA diagnostic code [DXCODE] and fee code (FEECODE=K122 or K123 or K704), where DXCODES = 295, 297, 298  b. CIHI-DAD/NACRS: DX10CODE1 F20 (excluding F20.4), F22-F25, F28-F29, F53.1; ICD-9: 295.x, 297.x, 298.x  c. OMHRS: Before 2016/17: AXIS1_DSM4CODE_DISCH1 = 295.x (all 295 codes), 297.x (all 297 codes), 298.x (all 298 codes); Provisional = 5; 2016/17-2019/20: DSM5CODE_DISCH1 = 293.81, 293.82, 295.x (all 295 codes), 297.x (all 297 codes), 298.x (all 298 codes), Provisional = 2 ; 2019/20 to present: ICD10CMCODE_DISCH1=F20.81, F20.9, F22, F23, F25, F06.0-F06.2, F28, F29; Provisional = 2. | CIHI-DAD, NACRS, OHIP, OMHRS | Psychiatric | 0.5% |
| Rheumatoid arthritis (29) | 3 physician visits (OHIP: 714) with 1 or more by a specialist (rheumatologist, internal medicine specialist, orthopedic surgeon) in a 2 year period or 1 ED visit or hospitalization (ICD-9: 714; ICD-10: M05, M06) | ORAD registry, CIHI-DAD, NACRS, OHIP | Autoimmune | 0.3% |
| Self-harm (21) | ≥1 ED visits or hospitalizations:  CIHI-DAD/NACRS: Before 2016/17: DX10CODE2-10 (NACRS)/DXCODE2-25(DAD) = X60-X84, Y10-Y19, Y28; 2017/18-2018/19: DX10CODE2-10 (NACRS)/DXCODE2-25(DAD) = X60-X84, Y10-Y19, Y28; 2019/20 to present: DX10CODE2-10 (NACRS)/DXCODE2-25(DAD) = X60-X84, Y10-Y19, Y28 | CIHI-DAD, NACRS | Psychiatric | 1.5% |
| Septal malformations (including patent foramen ovale) (30) | 1 physician visit (OHIP: 745) or 1 ED visit or hospitalization (ICD-9: 745.4, 745.5, 745.9; ICD-10: Q21) | CIHI-DAD, NACRS, OHIP | Cardiovascular | 0.2% |
| Somatoform disorder (31) | 2 physician visits (OHIP: 306) or 1 ED visit or hospitalization (ICD-9: 300.81, 300.82, 300.7, 306.4, 307.89, 306.8, 306.9; ICD-10: F45) | CIHI-DAD, NACRS, OHIP | Psychiatric | 0.3% |
| Stroke or transient ischemic attack (32) | 2 physician visits (OHIP: 432, 435, 436) or 1 ED visit or hospitalization (ICD-9: 362.3, 430, 431, 434.x, 435.x, 436; ICD-10: I60.x, I61.x, I63.x except I63.6, I64, H34.0, H34.1, G45.x except G45.4) | CIHI-DAD, NACRS, OHIP | Cerebrovascular | 0.2% |
| Substance use disorder (21) | ≥2 physician visits or ≥1 ED visits or hospitalizations:  a. Psychiatrist [SPEC=19] and outpatient (LOCATION: O, L, H, P) and non-lab service [substr(FEECODE,1,1) ne 'G'] OR Family physician / general practitioner / pediatrician [SPEC=00, SPEC=26] and mental illness or addiction diagnosis code [DXCODE] and outpatient (LOCATION: O, L, H, P) and non-lab service [substr(FEECODE,1,1) ne 'G'] OR pediatrician [SPEC=26] and undefined location (LOCATION=U) and MHA diagnostic code [DXCODE] and fee code (FEECODE=K122 or K123 or K704), where DXCODES = 291, 292, 303, 304  b. CIHI-DAD/NACRS: DX10CODE1 ICD-10: F10-F19, F55; ICD-9: 291.x (excluding 291.82), 292.x (excluding 292.85), 303.x, 304.x, 305.x  c. OMHRS: Before 2016/17: AXIS1_DSM4CODE_DISCH1 = 291.x (all 291 codes, excluding 291.82), 292.x (all 292 codes, excluding 292.85), 303.x (all 303 codes), 304.x (all 304 codes), 305.x (all 305 codes), PROVDX_DSM4CODE_ADM1: 4; 2016/17-2018/19: DSM5CODE_DISCH1 = 291.x (all 291 codes), 292.x (all 292 codes), 303.x (all 303 codes), 304.x (all 304 codes), 305.x, Provisional = 16; 2019/20 to present: ICD10CMCODE_DISCH1=F10-F19, Z72.0; Provisional = 16 | CIHI-DAD, NACRS, OHIP, OMHRS | Psychiatric | 3.8% |
| Systemic lupus erythematosus (33) | 3 physician visits (OHIP: 710) with ≥ 1 by a specialist (rheumatologist) or 1 hospital admission (ICD-9: 710.0; ICD-10: M32) | CIHI-DAD, NACRS, OHIP | Autoimmune | 0.2% |
| Thyroid disease (34) | 2 physician visits (OHIP: 242, 244) or 1 ED visit or hospitalization (ICD-9: 242, 244; ICD-10: E00, E03, E05) | CIHI-DAD, NACRS, OHIP | Metabolic | 4.4% |
| Traumatic brain injury (35) | ≥ 1 emergency department visits or hospitalizations (ICD-9: 800 (all 800 codes), 801 (all 801 codes), 803 (all 803 codes), 804 (all 804 codes), 850-854.1, 950.1-950.3; ICD-10: S02.0, S02.1, S02.8, S02.9, S04.0, S06.0-S06.9, S07.1) in **any** diagnosis field. | CIHI-DAD, NACRS, OHIP | Neurologic | 1.2% |
| Vertigo (36) | 1 ED visit or hospitalization (ICD-10: H81.1, H81.2, H81.3) | CIHI-DAD, NACRS | Neurologic | 0.4% |

Abbreviations: ADP = Assistive devices program; CIHI-DAD = Canadian Institute for Health Information-Discharge Abstract Database; CHF = congestive heart failure; COPD = chronic obstructive pulmonary disease; ED = emergency department; ICD = International Classification of Diseases; IPDB = ICES Physician database; NACRS = National Ambulatory Care Reporting System; OCCC = Ontario Crohn’s and Colitis Cohort dataset; ODD = Ontario Diabetes Dataset; OHIP = Ontario Health Insurance Plan; OMHRS = Ontario Mental Health Reporting System; OMID = Ontario Myocardial Infarction dataset; ORAD = Ontario Rheumatoid Arthritis database; SDS = Same Day Surgery database

a In analyses examining the effects of physical and psychiatric chronic conditions separately, neurodevelopmental conditions were grouped into the psychiatric disorder category

**Table S5.** Measurement of outcome variables

| **Variable** | **Disease/procedure** | **Codes** | **Incidence in cohort** |
| --- | --- | --- | --- |
| Severe maternal morbidity (SMM) | Acute renal failure | Acute renal failure: O90.4, N17, N19, N99.0  Dialysis: 1.PZ.21^^ | 0.05% |
|  | Cardiac conditions | Cardiac arrest and resuscitation: I46, I49.0, 1.HZ.09, 1.HZ.30  Cardiac complications of anesthesia: O74.2, O89.1  Cardiomyopathy: O90.3, I42, I43  Myocardial infarction: I21, I22  Pulmonary edema and heart failure: I50, J81 | 0.05% |
|  | Cerebrovascular accidents | Cerebral venous thrombosis in pregnancy, or in the puerperium: O22.5 or O87.3  Cerebrovascular diseases: subarachnoid and intracranial hemorrhage, cerebral infarction, stroke: I60-I64 | 0.02% |
|  | Embolism, shock, DIC | Disseminated intravascular coagulation: D65  Obstetric embolism: O88  Obstetric shock: O75.1, R57, T80.5, T88.6 | 0.08% |
|  | Sepsis | Puerperal sepsis: O85  Septicemia during labour: O75.3 | 0.21% |
|  | Severe hemorrhage | Antepartum hemorrhage with coagulation defect: O46.0  Curettage with RBC transfusion: (5.PC.91.GA or 5.PC.91.GC or 5.PC.91.GD) + BTREDBC=1 or Y^a^  Intrapartum hemorrhage with coagulation defect: O67.0  Intrapartum hemorrhage with RBC transfusion: O.67 + BTREDBC=1 or Y  Placental abruption with coagulation defect: O45.0  Placenta previa with hemorrhage with RBC transfusion: O44.1 + BTREDBC=1 or Y  Postpartum hemorrhage with RBC transfusion, procedures to the uterus or hysterectomy: O72 + (BTREDBC=1 or Y or [[1.RM.13^^ or 1.KT.51 or 5.PC.91.LA or 5.PC.91.HV] + BTREDBC=1 or Y] or [5.MD.60.RC, 5.MD.60.RD, 5.MD.60.KE, 5.MD.60.CB, 1.RM.89.LA] or [1.RM.87.LA-GX])   - Note: 1.RM.89.LA is included only if codes 1.PL.74, 1.RS.74 or 1.RS.80 are NOT also present | 0.46% |
|  | Severe uterine rupture | Rupture of the uterus with RBC transfusion, procedures to the uterus or hysterectomy: (O71.0 or O71.1) and (BTREDBC=1 or Y or [[1.RM.13^^ or 1.KT.51 or 5.PC.91.LA or 5.PC.91.HV + BTREDBC =1 or Y] or [5.MD.60.RC, 5.MD.60.RD, 5.MD.60.KE, 5.MD.60.CB, 1.RM.89.LA] or [1.RM.87.LA-GX])   - Note: 1.RM.89.LA is included only if codes 1.PL.74, 1.RS.74 or 1.RS.80 are NOT also present | 0.01% |
|  | SPE, HELLP, eclampsia | Severe preeclampsia and HELLP syndrome: O14.1, O14.2  Eclampsia: O15 | 0.29% |
|  | Assisted ventilation | Assisted ventilation through endotracheal tube: 1.GZ.31.CA-ND  Assisted ventilation through tracheostomy: 1.GZ.31.CR-ND | 0.07% |
|  | Hysterectomy | Hysterectomy: 5.MD.60.RC, 5.MD.60.RD, 5.MD.60.KE, 5.MD.60.CB, 1.RM.89.LA, 1.RM.87.LA-GX  Note: 1.RM.89.LA is included only if codes 1.PL.74, 1.RS.74 or 1.RS.80 are NOT also present | 0.15% |
|  | Maternal ICU admission | CIHI-DAD special care unit SCUadmdate1-6 variable | 0.29% |
|  | Surgical complications | Complications of obstetric surgery and procedures: O75.4  Evacuation of incisional hematoma with RBC transfusion: 5.PC.73.JS + BTREDBC=1 or Y  Reclosure of caesarean wound with RBC transfusion: (5.PC.80.JM or 5.PC.80.JH) + BTREDBC=1 or Y  Repair of bladder, urethra, or intestine: 5.PC.80.JR, 1.NK.80^^, 1.NM.80^^ | 0.11% |
|  | Other | Acute abdomen: K35, K37, K65, N73.3, N73.5  Acute fatty liver with red blood cell (RBC) or plasma transfusion: O26.6 + BTRED=1 or Y or BTPLASMA=1 or Y^b^  Acute psychosis: F53.1, F23  Adult respiratory distress syndrome: J80  Cerebral edema or coma: G93.6, R40.2  Hepatic failure: K71-K72  Pulmonary, cardiac, and CNS complications of anaesthesia during pregnancy, the puerperium, or labour and delivery: O29.0, O29.1, O29.2, O89.0, O89.1, O89.2, O74.0, O74.1, O74.2, O74.3  Sickle cell anemia with crisis: D57.0  Status asthmaticus: J45.01, J45.11, J45.81, J45.91  Status epilepticus: G41  Surgical or manual correction of inverted uterus for vaginal births only: 5.PC.91.HP, 5.PC.91.HQ (exclude cesarean births, 5.MD.60) | 0.18% |
| Maternal mortality | Death between conception and 42 days postpartum | Mother’s death date from RPDB and ORGD | 0.01% |
| Severe neonatal morbidity (0-27 days of life) | Gestational age < 32 weeks | MOMBABY: B_GESTWKS_DEL | 0.84% |
|  | Birthweight < 1500 grams | MOMBABY: B_WEIGHT | 0.73% |
|  | Birth trauma | ICD-10: P10.0 to P10.3, P13.0, P13.2, P13.3, P14.0, P14.1 P14.2, P14.3, P14.8, P14.9, P13.4 | 0.25% |
|  | Necrotising enterocolitis | ICD-10: P77 | 0.11% |
|  | Seizure | ICD-10: P90, R56 | 0.22% |
|  | Intraventricular haemorrhage (grade 2,3, or 4) | ICD-10: P52.1, P52.2 | 0.10% |
|  | Cerebral infarction | ICD-10: I63 | 0.01% |
|  | Periventricular leukomalacia | ICD-10: P91.2 | 0.02% |
|  | Neonatal encephalopathy | ICD-10: P91.5, P91.6, P91.8 | 0.25% |
|  | Infection | ICD-10: P36, G00-03, G05, A40, A41.5, A41.8, A41.9, B95.1, B96.2 |  |
|  | Respiratory distress syndrome | ICD-10: P22.0 | 1.30% |
|  | Pneumonia | ICD-10: P23; J12-18 | 0.24% |
|  | Chronic respiratory disease originating in the perinatal period | ICD-10: P27 | 0.16% |
|  | Other respiratory | ICD-10: P28.0; P28.5 | 1.27% |
|  | Perinatal intestinal perforation | ICD-10: P78.0 | 0.03% |
|  | Retinopathy of prematurity | ICD-10: H35.1 |  |
|  | Ventilatory support | CCI: 1GZ31CAEP, 1GZ31CAND, 1GZ31CAPK, 1GZ31CBND, 1GZ31CRND, 1GZ31GPND, 1GZ31JAMD, 1GZ31JANC, 1GZ31JAPK | 4.51% |
|  | Pneumothorax requiring intercostal catheter | CCI: 1GV52DA, 1GV52DATS, 1GV52HA, 1GV52HAHE, 1GV52HATK, 1GV52LA, 1GV52LATS, 1GV52LAXXE, 1GV54JATS, 1GV55JATS | 0.14% |
|  | Any body cavity surgical procedure | CCI: 1AA52, 1AA87, 1AC87, 1AE87, 1AF87, 1AG87, 1AJ87, 1AK87, 1AN52, 1AN59, 1AN87, 1AP59, 1AP72, 1AP87, 1AW59, 1AW72, 1AW87, 1AX87, 1BA72, 1BA80, 1BA87, 1BB72, 1BB80, 1BB87, 1BD72, 1BD80, 1BD87, 1BF80, 1BG72, 1BG80, 1BG87, 1BK59, 1BM72, 1BM80, 1BM87, 1BN72, 1BN80, 1BN87, 1BP72, 1BP80, 1BP87, 1BQ72, 1BQ80, 1BQ87, 1BS72, 1BS80, 1BS87, 1BT72, 1BT80, 1BT87, 1GA87, 1GA89, 1GB87, 1GB89, 1GD89, 1GE80, 1GE87, 1GE89, 1GE91, 1GH84, 1GJ86, 1GJ87, 1GK87, 1GK89, 1GM80, 1GM86, 1GM87, 1GN92, 1GR87, 1GR89, 1GR91, 1GT78, 1GT87, 1GT89, 1GT91, 1GV87, 1GV89, 1GW87, 1GX80, 1GX86, 1GX87, 1GY70, 1GY72, 1GY86, 1HJ76, 1HJ82, 1HN87, 1HP76, 1HP78, 1HP80, 1HP82, 1HP83, 1HP87, 1HR80, 1HR84, 1HR87, 1HS80 (excl. 1HS80G), 1HS90, 1HT80 (excl. 1HT80G), 1HT89, 1HT90, 1HU80 (excl. 1HU80G), 1HU90, 1HV80 (excl. 1HV80G), 1HV90, 1HW78, 1HW79, 1HX80, 1HX87, 1HX80, 1HZ87, 1IA76, 1IA80, 1IA87, 1IB76, 1IB79, 1IB80, 1IB82, 1IB87, 1IC76, 1IC80, 1IC82, 1IC87, 1ID76, 1ID80, 1ID82, 1ID86, 1ID87, 1IF83, 1IJ76, 1IJ80, 1IM76, 1IM80, 1IM82, 1IM83, 1IM87, 1IN83, 1IN84, 1IN87, 1JE57 (excl. 1JE57G), 1JE76, 1JE80, 1JE87, 1JJ76, 1JJ80, 1JK76, 1JK80, 1JK87, 1JW51 (excl. 1JW51G), 1JW57, 1JW76, 1LA84, 1LC84, 1LD84, 1NA72, 1NA74, 1NA76, 1NA77, 1NA80, 1NA84, 1NA86, 1NA87, 1NA88, 1NA89, 1NA90, 1NA91, 1NA92, 1NE80, 1NF76, 1NF78, 1NF80, 1NF82, 1NF84, 1NF86, 1NF87 (excl. 1NF87B), 1NF89, 1NF90, 1NF91, 1NF92, 1NK76, 1NK77, 1NK80, 1NK82, 1NK84, 1NK87 (excl. 1NK87B), 1NM74, 1NM76, 1NM77, 1NM80, 1NM82, 1NM87 (excl. 1NM87B), 1NM89, 1NM91, 1NP72, 1NP73, 1NP86, 1NQ74 (excl. 1NQ74B), 1NQ80, 1NQ84, 1NQ86, 1NQ87 (excl. 1NQ87B), 1NQ89, 1NQ90, 1NT80, 1NT84, 1NT86, 1NT87, 1NV89, 1OA87, 1OB87, 1OB89, 1OD76, 1OD89, 1OE76, 1OE80, 1OE89, 1OJ76 (excl. 1OJ76B), 1OJ87, 1OJ89, 1OK87, 1OK89, 1OK91, 1OT72, 1OT87, 1OT91, 1PB87, 1PB89, 1PC80, 1PC87 (excl. 1PC87D), 1PC89, 1PC91, 1PE57 (excl. 1PE57BD), 1PE80 (excl. 1PE80D), 1PE82, 1PE87 (excl. 1PE87D), 1PE89 (excl. 1PE89D), 1PG76, 1PG77, 1PG80 (excl.1PG80D), 1PG86, 1PG89, 1PL74 (excl.1PL74CD), 1PL80, 1PM79, 1PM86, 1PM87 (excl. 1PM87B), 1PM89, 1PM90, 1PM91, 1PM92, 1QE53, 1QE80, 1QE82, 1QE84, 1QE87, 1QE89, 1QG89, 1QM74, 1QM80, 1QM87, 1QM89, 1QM91, 1QN82, 1QT87, 1QT91, 1RB74, 1RB80, 1RB83, 1RB87, 1RB89, 1RD89, 1RF51, 1RF72, 1RF74, 1RF80, 1RF87, 1RF89, 1RM87 (excl.1RM87B), 1RM89, 1RM91, 1RN87, 1RN89, 1RS74, 1RS80, 1RS86, 1RS87, 1RS89, 1RW87, 1RW88, 1RW91, 1RW92, 1SA74, 1SA75, 1SA80, 1SA89, 1SC74, 1SC75, 1SC80, 1SC87, 1SC89, 1SE53, 1SE89 (excl. 1SE89D), 1SF80, 1SF87, 1SF89, 1SG80, 1SG87, 1SH87, 1SM74, 1SM80, 1SM87, 1SN87, 1SN93, 1SQ53, 1SQ74, 1SQ80, 1SQ87, 1SQ91, 1SQ93, 1SW74, 1SY80, 1SY84, 1SY87, 1SZ87, 1VA53, 1VA74, 1VA75, 1VA80, 1VA87, 1VA93, 1VC74, 1VC80, 1VC87, 1VC91, 1VC93, 1VE80, 1VG53, 1VG55, 1VG72, 1VG73, 1VG74, 1VG75, 1VG80, 1VG87, 1VG93, 1VK80, 1VK87, 1VK89, 1VL80, 1VL87, 1VM80, 1VM87, 1VN80, 1VN87, 1VP74, 1VP80, 1VP87, 1VP89, 1VQ74, 1VQ79, 1VQ80, 1VQ82, 1VQ87, 1VQ91, 1VQ93, 1VS72, 1VS80, 1VX87 | 0.28% |
|  | Resuscitation by intubation and/or chest compressions | CCI: 1.GZ.30^^, 1.HZ.30^^ | 0.22% |
|  | Central venous or arterial catheter insertion | CCI: 1KV53HACH, 1KV53HAFT, 1KV53LAFT, 2IM28GP, 2LZ28GQPL, 2LZ28GRPL, 2LZ28JAPL, 1KX53HACH, 1KX53HAFT*, 1KX53LAFT*, 2LZ28GQPL, 2LZ28GRPL, 1IS53^^ *Up until 2015 umbilical venous catherization (UVC) was classified in these codes. After 2015, they were found in 1IS53^^. | 1.5% |
|  | Administration of any intravenous fluid | CCI: 1.LZ.35^^ | 1.92% |
|  | Transfusion of red blood cells or a blood product | CCI: 1LZ19HHU1A, 1LZ19HHU1J, 1LZ19HHU2A, 1LZ19HHU2J, 1LZ19HHU3J, 1LZ19HHU4J, 1LZ19HHU5J, 1LZ19HHU6A, 1LZ19HHU6J, 1LZ19HHU9A, 1LZ19HHU9J, 1LZ19HMU1, 1LZ19HMU2, 1LZ19HMU9, 1LZ35HAC5. Note: CIHI has a “BLDTRNSF” variable which also summarizes all the above. | 0.01% |
| Neonatal or in-hospital death | All-cause neonatal mortality before 28 days of life, or any time before hospital discharge | ICD-10: O96 or other all-cause neonatal mortality in RPDB | 0.19% |

Abbreviations: CCI = Canadian Classification of Health Interventions; CCP = Canadian Classification of Diagnostic, Therapeutic, and Surgical Procedures; ICD = International Classification of Diseases and Related Health Problems; OHIP = Ontario Health Insurance Plan; RPDB = Registered Persons Database.

a BTRED = ‘Y’ prior to FY2011 and BTRED = ‘1’ from FY2011 on

b BTPLASMA = ‘Y’ prior to FY2011 and BTPLASMA = ‘1’ from FY2011 on

**Table S6.** Measurement of covariates

| **Covariate** | **Definition** | **Dataset** |
| --- | --- | --- |
| Age | Mother’s age at conception | MOMBABY |
| Year of conception | Year of pregnancy conception (categorical in 4-year groupings starting with 2007-2010) | MOMBABY |
| Parity | Number of prior obstetrical deliveries (defined at conception) | MOMBABY |
| Neighbourhood income quintile | Median family income in dissemination area associated  with residential postal code | RPDB linked with Census data |
| Rural/remote residence | Residence in an area with a population <10,000 based on postal code, as of the index pregnancy conception date | RPDB pstlcode variable linked with Census data |
| Immigrant/refugee status | Immigrated to Canada from 1985 onward (inception of database) classified as immigrants; else, classified as long-term residents | CIC (IRCC Permanent Residents Database) |
| Recent history of interpersonal violence | History of interpersonal violence in ≥1 emergency department visits or hospital admissions for physical, sexual, or psychological violence in the 2 years before conception (ICD-10: X85 to Y09) | CIHI-DAD/NACRS |
| Infant sex^a^ | Sex assigned to the infant at birth (male or female) | b_sex variable in MOMBABY |
| Multiple birth status^a^ | Multiple birth variable | MOMBABY |
| Adverse childhood experiences^b^ | Traumatic events occurring before the age of 18 that could have long-term effects on health and wellbeing (e.g., abuse, neglect, household dysfunction, mental illness in the household) (37) | CIHI-DAD, NACRS, OHIP, OMHRS, RPDB |

Abbreviations: CIC = Citizenship and Immigration Canada; CIHI-DAD = Canadian Institute for Health Information Discharge Abstract Database; ICD = International Classification of Diseases and Related Health Problems; IRCC = Immigration, Refugees and Citizenship Canada; NACRS = National Ambulatory Care Reporting System; OHIP = Ontario Health Insurance Plan; OMHRS = Ontario Mental Health Reporting System; RPDB = Registered Persons Database.

a Known for pregnancies ending in a live birth or stillbirth only

b Measured for individuals born in Ontario in 1988 or later with a valid linkage to their mother

**Table S7.** Comparison of characteristics among pregnancies to individuals with migraine and ≥ 1 chronic condition, migraine alone, and other chronic condition(s) alone, compared with pregnancies to individuals with neither migraine nor other chronic conditions, Ontario, Canada, 2007-2022.

|  | **Migraine and ≥ 1 other chronic condition** | | **Migraine alone** | | **Other chronic condition(s) alone** | | **No migraine/other chronic conditions** |
| --- | --- | --- | --- | --- | --- | --- | --- |
|  | *n* = 179,461  (6.8%) | *d* ^a^ | *n* = 83,652  (3.2%) | *d* | *n* = 1,204,286 (45.7%) | *d* | *n* = 1,166,436  (44.3%) |
| Age at conception |  |  |  |  |  |  |  |
| 13-24 years | 39,559 (22.0) | 0.08 | 16,309 (19.5) | 0.02 | 263,159 (21.9) | 0.07 | 219,997 (18.9) |
| 25-34 years | 102,631 (57.2) | 0.07 | 51,983 (62.1) | 0.04 | 668,052 (55.5) | 0.10 | 704,826 (60.4) |
| 35-44 years | 36,230 (20.2) | 0.00 | 15,086 (18.0) | 0.06 | 266,600 (22.1) | 0.04 | 237,471 (20.4) |
| 45-54 years | 1041 (0.6) | 0.03 | 274 (0.3) | 0.00 | 6475 (0.5) | 0.03 | 4142 (0.4) |
| Year of conception |  |  |  |  |  |  |  |
| 2007-2010 | 47,429 (26.4) | 0.01 | 23,146 (27.7) | 0.02 | 305,665 (25.4) | 0.04 | 314,969 (27.0) |
| 2011-2014 | 47,829 (26.7) | 0.01 | 22,060 (26.4) | 0.02 | 328,584 (27.3) | 0.00 | 316,040 (27.1) |
| 2015-2018 | 47,716 (26.6) | 0.03 | 21,260 (25.4) | 0.00 | 325,470 (27.0) | 0.04 | 293,909 (25.2) |
| 2019-2022 | 36,487 (20.3) | 0.01 | 17,186 (20.5) | 0.00 | 244,567 (20.3) | 0.01 | 241,518 (20.7) |
| Multiparous | 104,627 (58.3) | 0.13 | 43,590 (52.1) | 0.01 | 687,160 (57.1) | 0.11 | 603,636 (51.8) |
| Pregnancy outcome |  |  |  |  |  |  |  |
| Livebirth | 112,768 (62.8) | 0.10 | 55,900 (66.8) | 0.02 | 768,705 (63.8) | 0.08 | 787,903 (67.6) |
| Stillbirth | 746 (0.4) | 0.02 | 268 (0.3) | 0.00 | 4865 (0.4) | 0.00 | 3574 (0.3) |
| Miscarriage^b^ | 29,942 (16.7) | 0.11 | 11,462 (13.7) | 0.03 | 183,370 (15.2) | 0.03 | 149,221 (12.8) |
| Induced abortion | 36,005 (20.1) | 0.02 | 16,022 (19.2) | 0.01 | 247,346 (20.5) | 0.01 | 225,738 (19.4) |
| Neighbourhood  income quintile (Q) |  |  |  |  |  |  |  |
| Q1 (lowest) | 45,709 (25.5) | 0.11 | 17,711 (21.2) | 0.01 | 291,073 (24.2) | 0.08 | 243,509 (20.9) |
| Q2 | 38,533 (21.5) | 0.03 | 17,000 (20.3) | 0.00 | 250,113 (20.8) | 0.01 | 236,662 (20.3) |
| Q3 | 36,863 (20.5) | 0.01 | 17,856 (21.4) | 0.01 | 246,215 (20.4) | 0.01 | 244,212 (20.9) |
| Q4 | 33,605 (18.7) | 0.06 | 17,640 (21.1) | 0.00 | 233,956 (19.4) | 0.04 | 243,560 (20.9) |
| Q5 (highest) | 24,751 (13.8) | 0.09 | 13,445 (16.1) | 0.02 | 182,929 (15.2) | 0.05 | 198,493 (17.0) |
| Rural or remote  residence | 17,320 (9.7) | 0.02 | 7,317 (8.8) | 0.01 | 118,437 (9.8) | 0.02 | 106,665 (9.1) |
| Immigration status |  |  |  |  |  |  |  |
| Long-term resident | 140,619 (78.4) | 0.09 | 59,879 (71.6) | 0.07 | 944,357 (78.4) | 0.09 | 818,603 (70.2) |
| Immigrant | 30,156 (16.8) | 0.11 | 19,367 (23.2) | 0.05 | 212,896 (17.7) | 0.09 | 297,241 (25.5) |
| Refugee | 8696 (4.8) | 0.03 | 4406 (5.3) | 0.05 | 47,033 (3.9) | 0.02 | 50,592 (4.3) |
| Recent history of interpersonal violence | 2934 (1.6) | 0.13 | 378 (0.5) | 0.01 | 16,721 (1.4) | 0.11 | 4535 (0.4) |
| Adverse childhood experiences^c,d^ | 10,173 (32.7) | 0.30 | 2470 (20.5) | 0.02 | 65,460 (32.2) | 0.28 | 30,855 (19.8) |

a Standardized difference *d* > 0.10 represents a meaningful imbalance between the groups (1)

b Threatened abortion early in pregnancy without a subsequent pregnancy outcome was used as a proxy for miscarriage occurring outside the healthcare system (see Supplemental Table S3)

c Measured when the individual was aged 0-17 years

d Denominator is n=402,425 individuals who were born in Ontario in 1988 or later and had a valid linkage to their mother

Note: All characteristics are reported as n (%)

**Table S8.** Estimates of RR among “migraine alone” group adjusted for bias due to migraine misclassification.

|  | SMM-M | | RR  [95% CI] | SNM-M | | RR  [95% CI] |
| --- | --- | --- | --- | --- | --- | --- |
|  | N+ | N- |  | N+ | N- |  |
| Migraine  No other chronic conditions | 788.9 | 11,192.5 | 5.23  [4.88-5.60] | 1316.9 | 3828.9 | 4.27  [4.07-4.48] |
| No migraine  No other chronic conditions | 15,599.1 | 1,238,106.6 | [ref] | 49231.1 | 822,313.2 | [ref] |

Abbreviations: RR = relative risk. SMM-M = severe maternal morbidity or mortality. SNM-M = severe neonatal morbidity or mortality.

Note: The example in this table illustrates the possible impact of bias due to migraine misclassification on estimates of RR among those with migraine alone on the composite outcomes, SMM-M and SNM-M. Estimates of migraine sensitivity (26.5%) and specificity (93.5%) used to produce these bias-adjusted estimates were obtained from Albanese et al. *Epidemiology*. 2025; 36(5), 599-605. (3)

Simple bias-adjusted estimates were obtained using the worksheet provided by Fox et al., 2021. (38)

**Table S9.** Additive interaction between migraine in the 5 years before conception and type of chronic condition (grouped by primary body system affected) on the risk of SMM-M

| Risk Factor | N (%) with outcome | RR  (95% CI) | aRR  (95% CI) | aRERI  (95% CI) | aAP (%)  (95% CI) |
| --- | --- | --- | --- | --- | --- |
| *Autoimmune disease* |  |  |  |  |  |
| Migraine + autoimmune disease | 209 (2.6) | 1.66 (1.45-1.91) | 1.45 (1.26-1.66) | 0.04 (-0.16,0.26) | 2.74 (-13.9,14.9) |
| Migraine alone | 4743 (1.9) | 1.22 (1.18-1.26) | 1.15 (1.12-1.19) |  |  |
| Autoimmune disease alone | 1161 (2.1) | 1.35 (1.27-1.43) | 1.26 (1.19-1.33) |  |  |
| No migraine/autoimmune disease | 35,095 (1.5) | 1.00 (ref) | 1.00 (ref) |  |  |
| *Cardiovascular disease* |  |  |  |  |  |
| Migraine + cardiovascular disease | 450 (4.1) | 2.71 (2.46-2.98) | 2.25 (2.05-2.47) | 0.11 (-0.11,0.35) | 5.04 (-4.72,14.8) |
| Migraine alone | 4502 (1.8) | 1.20 (1.16-1.24) | 1.14 (1.11-1.18) |  |  |
| Cardiovascular disease alone | 1926 (3.5) | 2.30 (2.19-2.41) | 1.99 (1.90-2.09) |  |  |
| No migraine/cardiovascular disease | 34,330 (1.5) | 1.00 (ref) | 1.00 (ref) |  |  |
| *Cerebrovascular disease* |  |  |  |  |  |
| Migraine + cerebrovascular disease | 90 (3.6) | 2.23 (1.81-2.76) | 1.88 (1.52-2.32) | -0.32 (-0.86,0.22) | -17.1 (-54.8,8.53) |
| Migraine alone | 4862 (1.9) | 1.22 (1.18-1.25) | 1.15 (1.11-1.18) |  |  |
| Cerebrovascular disease alone | 118 (4.0) | 2.48 (2.05-3.01) | 2.05 (1.69-2.49) |  |  |
| No migraine/cerebrovascular disease | 36,138 (1.5) | 1.00 (ref) | 1.00 (ref) |  |  |
| *Gastrointestinal disease* |  |  |  |  |  |
| Migraine + gastrointestinal disease | 156 (2.5) | 1.62 (1.39-1.90) | 1.40 (1.19-1.63) | 0.08 (-0.15,0.34) | 5.99 (-13.8,19.7) |
| Migraine alone | 4796 (1.9) | 1.22 (1.18-1.25) | 1.15 (1.12-1.19) |  |  |
| Gastrointestinal disease alone | 494 (1.9) | 1.26 (1.16-1.38) | 1.16 (1.06-1.27) |  |  |
| No migraine/gastrointestinal disease | 35,762 (1.5) | 1.00 (ref) | 1.00 (ref) |  |  |
| *Metabolic disease* |  |  |  |  |  |
| Migraine + metabolic disease | 1250 (2.5) | 1.74 (1.65-1.85) | 1.56 (1.47-1.65) | 0.03 (-0.07,0.13) | 1.86 (-4.82,7.79) |
| Migraine alone | 3702 (1.7) | 1.21 (1.17-1.25) | 1.15 (1.11-1.19) |  |  |
| Metabolic disease alone | 7777 (2.1) | 1.46 (1.42-1.50) | 1.38 (1.34-1.42) |  |  |
| No migraine/metabolic disease | 28,479 (1.4) | 1.00 (ref) | 1.00 (ref) |  |  |
| *Neurodevelopmental disorders* |  |  |  |  |  |
| Migraine + neurodevelopmental disorder | 159 (2.2) | 1.40 (1.20-1.64) | 1.30 (1.11-1.53) | 0.12 (-0.01,0.35) | 9.07 (-9.56,21.7) |
| Migraine alone | 4793 (1.9) | 1.22 (1.18-1.26) | 1.15 (1.11-1.19) |  |  |
| Neurodevelopmental disorder alone | 716 (1.6) | 1.05 (0.97-1.13) | 1.03 (0.96-1.11) |  |  |
| No migraine/ neurodevelopmental disorder | 35,540 (1.5) | 1.00 (ref) | 1.00 (ref) |  |  |
| *Neurological disease* |  |  |  |  |  |
| Migraine + other neurological disease | 321 (2.9) | 1.86 (1.66-22.08) | 1.60 (1.43-1.79) | -0.07 (-0.24,0.11) | -5.34 (-20.3,6.44) |
| Migraine alone | 4631 (1.8) | 1.20 (1.17-1.24) | 1.14 (1.11-1.18) |  |  |
| Other neurological disease alone | 889 (2.2) | 1.42 (1.33-1.51) | 1.29 (1.21-1.38) |  |  |
| No migraine/other neurological disease | 35,367 (1.5) | 1.00 (ref) | 1.00 (ref) |  |  |
| *Pain syndromes* |  |  |  |  |  |
| Migraine + other pain syndrome | 1196 (2.3) | 1.51 (1.42-1.60) | 1.35 (1.27-1.43) | 0.06 (-0.03,0.16) | 4.63 (-2.38,10.8) |
| Migraine alone | 3756 (1.8) | 1.19 (1.15-1.23) | 1.14 (1.10-1.18) |  |  |
| Other pain syndrome alone | 4495 (1.8) | 1.22 (1.19-1.26) | 1.15 (1.11-1.18) |  |  |
| No migraine/other pain syndrome | 31,761 (1.5) | 1.00 (ref) | 1.00 (ref) |  |  |
| *Psychiatric disorders* |  |  |  |  |  |
| Migraine + psychiatric disorder | 2516 (2.1) | 1.41 (1.36-1.47) | 1.33 (1.28-1.39) | 0.07 (-0.01,0.14) | 5.06 (-0.50,10.2) |
| Migraine alone | 2436 (1.7) | 1.16 (1.12-1.21) | 1.13 (1.09-1.18) |  |  |
| Psychiatric disorder alone | 11,799 (1.7) | 1.15 (1.13-1.18) | 1.13 (1.11-1.16) |  |  |
| No migraine/psychiatric disorder | 24,457 (1.5) | 1.00 (ref) | 1.00 (ref) |  |  |
| *Respiratory disease* |  |  |  |  |  |
| Migraine + respiratory disease | 661 (2.3) | 1.49 (1.38-1.61) | 1.35 (1.25-1.46) | 0.06 (-0.06,0.18) | 4.19 (-4.94,11.8) |
| Migraine alone | 4291 (1.8) | 1.21 (1.17-1.25) | 1.14 (1.11-1.18) |  |  |
| Respiratory disease alone | 2858 (1.8) | 1.19 (1.15-1.24) | 1.15 (1.11-1.20) |  |  |
| No migraine/respiratory disease | 33,398 (1.5) | 1.00 (ref) | 1.00 (ref) |  |  |
| *Sleep disorders* |  |  |  |  |  |
| Migraine + sleep disorder | 15 (3.6) | 2.27 (1.37-3.75) | 1.60 (0.99-2.63) | 0.10 (-0.65,1.20) | 6.44 (-7.91,32.5) |
| Migraine alone | 4937 (1.9) | 1.22 (1.19-1.26) | 1.15 (1.12-1.19) |  |  |
| Sleep disorder alone | 55 (3.0) | 1.90 (1.46-2.47) | 1.34 (1.03-1.74) |  |  |
| No migraine/sleep disorder | 36,201 (1.5) | 1.00 (ref) | 1.00 (ref) |  |  |

Abbreviations: aAP = adjusted attributable proportion due to interaction; aRERI = adjusted relative excess risk due to interaction; aRR = adjusted relative risk; CI = confidence interval; RR = relative risk; SMM-M = severe maternal morbidity or mortality.

Note: aRR, aRERI, and aAP adjusted for age, parity, neighbourhood income quintile, rural residence, immigrant/refugee status, recent history of interpersonal violence, other physical chronic conditions, and other psychiatric or neurodevelopmental chronic conditions.

**Table S10.** Additive interaction between migraine in the 5 years before conception and type of chronic conditions (grouped by primary body system affected) on the risk of SNM-M.

| Risk Factor | N (%) with outcome | RR  (95% CI) | aRR  (95% CI) | aRERI  (95% CI) | aAP (%)  (95% CI) |
| --- | --- | --- | --- | --- | --- |
| *Autoimmune disease* |  |  |  |  |  |
| Migraine + autoimmune disease | 487 (9.2) | 1.32 (1.21-1.43) | 1.18 (1.09-1.29) | 0.01 (-0.10,0.12) | 0.49 (-10.2,9.03) |
| Migraine alone | 12,649 (7.9) | 1.13 (1.11-1.15) | 1.07 (1.05-1.08) |  |  |
| Autoimmune disease alone | 3028 (8.1) | 1.16 (1.12-1.20) | 1.11 (1.07-1.15) |  |  |
| No migraine/autoimmune disease | 103,930 (7.0) | 1.00 (ref) | 1.00 (ref) |  |  |
| *Cardiovascular disease* |  |  |  |  |  |
| Migraine + cardiovascular disease | 790 (12.0) | 1.71 (1.60-1.83) | 1.56 (1.46-1.67) | 0.03 (-0.09,15.2) | 1.85 (-6.33,8.88) |
| Migraine alone | 12,346 (7.8) | 1.12 (1.10-1.14) | 1.06 (1.04-1.08) |  |  |
| Cardiovascular disease alone | 3676 (10.8) | 1.53 (1.49-1.58) | 1.47 (1.42-1.51) |  |  |
| No migraine/cardiovascular disease | 103,282 (6.9) | 1.00 (ref) | 1.00 (ref) |  |  |
| *Cerebrovascular disease* |  |  |  |  |  |
| Migraine + cerebrovascular disease | 159 (10.8) | 1.53 (1.31-1.78) | 1.37 (1.18-1.59) | 0.14 (-0.14,0.43) | 10.5 (-12.2,26.6) |
| Migraine alone | 12,977 (7.9) | 1.13 (1.11-1.15) | 1.06 (1.05-1.08) |  |  |
| Cerebrovascular disease alone | 159 (9.2) | 1.30 (1.11-1.51) | 1.16 (1.00-1.35) |  |  |
| No migraine/cerebrovascular disease | 106,799 (7.0) | 1.00 (ref) | 1.00 (ref) |  |  |
| *Gastrointestinal disease* |  |  |  |  |  |
| Migraine + gastrointestinal disease | 372 (9.3) | 1.32 (1.20-1.45) | 1.13 (1.02-1.24) | 0.05 (-0.07,0.18) | 4.40 (-8.08,14.0) |
| Migraine alone | 12,764 (7.9) | 1.13 (1.11-1.15) | 1.07 (1.05-1.08) |  |  |
| Gastrointestinal disease alone | 1311 (7.8) | 1.10 (1.05-1.16) | 1.01 (0.96-1.07) |  |  |
| No migraine/gastrointestinal disease | 105,647 (7.0) | 1.00 (ref) | 1.00 (ref) |  |  |
| *Metabolic disease* |  |  |  |  |  |
| Migraine + metabolic disease | 3233 (9.9) | 1.47 (1.42-1.52) | 1.35 (1.31-1.40) | 0.02 (-0.04,0.07) | 1.12 (-3.01,4.95) |
| Migraine alone | 9903 (7.5) | 1.12 (1.10-1.14) | 1.07 (1.04-1.09) |  |  |
| Metabolic disease alone | 21,935 (8.8) | 1.30 (1.28-1.32) | 1.27 (1.25-1.29) |  |  |
| No migraine/metabolic disease | 85,023 (6.7) | 1.00 (ref) | 1.00 (ref) |  |  |
| *Neurodevelopmental disorders* |  |  |  |  |  |
| Migraine + neurodevelopmental disorder | 489 (11.3) | 1.61 (1.48-1.76) | 1.32 (1.22-1.44) | 0.13 (0.00,0.26) | 9.57 (-0.27,17.5) |
| Migraine alone | 12,647 (7.9) | 1.12 (1.10-1.14) | 1.06 (1.04-1.08) |  |  |
| Neurodevelopmental disorder alone | 2345 (9.3) | 1.34 (1.28-1.39) | 1.14 (1.09-1.18) |  |  |
| No migraine/neurodevelopmental disorder | 104,613 (7.0) | 1.00 (ref) | 1.00 (ref) |  |  |
| *Neurological disease* |  |  |  |  |  |
| Migraine + other neurological disease | 668 (10.2) | 1.44 (1.34-1.55) | 1.18 (1.09-1.27) | -0.03 (-0.13,0.08) | -2.15 (-11.9,5.94) |
| Migraine alone | 12,468 (7.9) | 1.12 (1.10-1.14) | 1.07 (1.05-1.09) |  |  |
| Other neurological disease alone | 2223 (9.3) | 1.32 (1.27-1.38) | 1.14 (1.09-1.19) |  |  |
| No migraine/other neurological disease | 104,735 (7.0) | 1.00 (ref) | 1.00 (ref) |  |  |
| *Pain syndromes* |  |  |  |  |  |
| Migraine + other pain syndrome | 2850 (9.0) | 1.28 (1.24-1.33) | 1.17 (1.13-1.21) | 0.04 (-0.01,0.10) | 3.68 (-0.64,7.99) |
| Migraine alone | 10,286 (7.7) | 1.11 (1.09-1.13) | 1.06 (1.04-1.08) |  |  |
| Other pain syndrome alone | 11,689 (7.7) | 1.11 (1.09-1.13) | 1.07 (1.05-1.09) |  |  |
| No migraine/other pain syndrome | 95,269 (6.9) | 1.00 (ref) | 1.00 (ref) |  |  |
| *Psychiatric disorders* |  |  |  |  |  |
| Migraine + psychiatric disorder | 6585 (9.1) | 1.39 (1.36-1.43) | 1.33 (1.30-1.36) | 0.03 (-0.01,0.07) | 2.16 (-1.18,5.33) |
| Migraine alone | 6551 (7.0) | 1.08 (1.05-1.11) | 1.06 (1.03-1.09) |  |  |
| Psychiatric disorder alone | 34,748 (8.3) | 1.27 (1.25-1.29) | 1.24 (1.23-1.26) |  |  |
| No migraine/psychiatric disorder | 72,210 (6.5) | 1.00 (ref) | 1.00 (ref) |  |  |
| *Respiratory disease* |  |  |  |  |  |
| Migraine + respiratory disease | 1589 (9.0) | 1.29 (1.23-1.35) | 1.16 (1.10-1.21) | 0.00 (-0.06,0.07) | 0.41 (-5.55,5.68) |
| Migraine alone | 11,547 (7.8) | 1.12 (1.10-1.15) | 1.06 (1.04-1.08) |  |  |
| Respiratory disease alone | 7753 (8.0) | 1.14 (1.12-1.17) | 1.09 (1.06-1.11) |  |  |
| No migraine/respiratory disease | 99,205 (7.0) | 1.00 (ref) | 1.00 (ref) |  |  |
| *Sleep disorders* |  |  |  |  |  |
| Migraine + sleep disorder | 46 (17.1) | 2.41 (1.85-3.13) | 1.81 (1.39-2.36) | 0.15 (-0.35,0.76) | 8.70 (-30.9,29.8) |
| Migraine alone | 13,090 (7.9) | 1.13 (1.11-1.15) | 1.07 (1.05-1.08) |  |  |
| Sleep disorder alone | 171 (14.1) | 1.99 (1.73-2.29) | 1.55 (1.35-1.78) |  |  |
| No migraine/sleep disorder | 106,787 (7.0) | 1.00 (ref) | 1.00 (ref) |  |  |

Abbreviations: aAP = adjusted attributable proportion due to interaction; aRERI = adjusted relative excess risk due to interaction; aRR = adjusted relative risk; CI = confidence interval; RR = relative risk; SNM-M = severe neonatal morbidity or mortality.

Note: aRR, aRERI, and aAP adjusted for age, parity, neighbourhood income quintile, rural residence, immigrant/refugee status, recent history of interpersonal violence, fetal sex, other physical chronic conditions, and other psychiatric or neurodevelopmental chronic conditions.

**Table S11.** Additive interaction between migraine in the 5 years before conception and any other chronic conditions on the risk of individual indicators of SMM.

| Risk Factor | N (%) with outcome | RR  (95% CI) | aRR  (95% CI) | aRERI  (95% CI) | aAP (%)  (95% CI) |
| --- | --- | --- | --- | --- | --- |
| **Acute renal failure** |  |  |  |  |  |
| Migraine + ≥ 1 other chronic condition | 121 (0.1) | 1.89 (1.54-2.31) | 1.88 (1.53-2.31) | 0.02 (-0.57,0.52) | 0.80 (-34.0,23.9) |
| Migraine alone | 32 (0.0) | 1.09 (0.76-1.56) | 1.11 (0.78-1.58) |  |  |
| Other chronic condition(s) alone | 766 (0.1) | 1.79 (1.59-2.02) | 1.76 (1.56-1.98) |  |  |
| No migraine/other chronic condition | 410 (0.0) | 1.00 (ref) | 1.00 (ref) |  |  |
| **Cardiac conditions** |  |  |  |  |  |
| Migraine + ≥ 1 other chronic condition | 148 (0.1) | 2.50 (2.07-3.03) | 2.49 (2.05-3.02) | 0.65 (0.02,1.22) | 26.2 (0.34,43.5) |
| Migraine alone | 29 (0.0) | 1.08 (0.75-1.57) | 1.10 (0.76-1.60) |  |  |
| Other chronic condition(s) alone | 700 (0.1) | 1.76 (1.55-2.00) | 1.74 (1.53-1.97) |  |  |
| No migraine/other chronic condition | 374 (0.0) | 1.00 (ref) | 1.00 (ref) |  |  |
| **Cerebrovascular accidents** |  |  |  |  |  |
| Migraine + ≥ 1 other chronic condition | 73 (0.0) | 3.51 (2.63-4.69) | 3.46 (2.59-4.63) | 0.09 (-1.49,1.39) | 2.69 (-4.75,3.40) |
| Migraine alone | 27 (0.0) | 2.85 (1.89-4.30) | 2.90 (1.92-4.38) |  |  |
| Other chronic condition(s) alone | 204 (0.0) | 1.50 (1.20-1.86) | 1.47 (1.18-1.84) |  |  |
| No migraine/other chronic condition | 131 (0.0) | 1.00 (ref) | 1.00 (ref) |  |  |
| **Embolism, shock, DIC** |  |  |  |  |  |
| Migraine + ≥ 1 other chronic condition | 188 (0.1) | 1.68 (1.43-1.98) | 1.63 (1.39-1.92) | 0.02 (-0.42,0.40) | 1.00 (-27.8,22.0) |
| Migraine alone | 66 (0.1) | 1.27 (0.99-1.63) | 1.28 (0.99-1.64) |  |  |
| Other chronic condition(s) alone | 1033 (0.1) | 1.38 (1.26-1.52) | 1.34 (1.21-1.47) |  |  |
| No migraine/other chronic condition | 722 (0.1) | 1.00 (ref) | 1.00 (ref) |  |  |
| **Sepsis** |  |  |  |  |  |
| Migraine + ≥ 1 other chronic condition | 475 (0.3) | 1.48 (1.34-1.64) | 1.51 (1.37-1.67) | 0.02 (-0.22,0.24) | 1.43 (-15.3,15.1) |
| Migraine alone | 172 (0.2) | 1.16 (0.99-1.35) | 1.16 (0.99-1.36) |  |  |
| Other chronic condition(s) alone | 2810 (0.2) | 1.31 (1.24-1.39) | 1.33 (1.26-1.41) |  |  |
| No migraine/other chronic condition | 2073 (0.2) | 1.00 (ref) | 1.00 (ref) |  |  |
| **Severe hemorrhage** |  |  |  |  |  |
| Migraine + ≥ 1 other chronic condition | 960 (0.5) | 1.24 (1.16-1.33) | 1.24 (1.16-1.33) | 0.12 (-0.02,0.25) | 9.65 (-1.79,19.6) |
| Migraine alone | 356 (0.4) | 0.99 (0.89-1.10) | 1.00 (0.90-1.11) |  |  |
| Other chronic condition(s) alone | 5851 (0.5) | 1.13 (1.09-1.17) | 1.12 (1.08-1.16) |  |  |
| No migraine/other chronic condition | 5014 (0.4) | 1.00 (ref) | 1.00 (ref) |  |  |
| **Severe uterine rupture** |  |  |  |  |  |
| Migraine + ≥ 1 other chronic condition | 37 (0.0) | 2.15 (1.48-3.11) | 2.01 (1.38-2.92) | 0.58 (-0.66,1.56) | 29.0 (-39.2,60.7) |
| Migraine alone | 9 (0.0) | 1.12 (0.57-2.21) | 1.14 (0.58-2.24) |  |  |
| Other chronic condition(s) alone | 159 (0.0) | 1.38 (1.08-1.75) | 1.29 (1.01-1.64) |  |  |
| No migraine/other chronic condition | 112 (0.0) | 1.00 (ref) | 1.00 (ref) |  |  |
| **SPE, HELLP, eclampsia** |  |  |  |  |  |
| Migraine + ≥ 1 other chronic condition | 720 (0.4) | 1.68 (1.54-1.82) | 1.76 (1.62-1.91) | 0.15 (-0.06,0.35) | 8.56 (-4.00,19.23) |
| Migraine alone | 240 (0.3) | 1.21 (1.06-1.38) | 1.23 (1.08-1.41) |  |  |
| Other chronic condition(s) alone | 3855 (0.3) | 1.34 (1.28-1.41) | 1.38 (1.31-1.44) |  |  |
| No migraine/other chronic condition | 2765 (0.2) | 1.00 (ref) | 1.00 (ref) |  |  |
| **Assisted ventilation** |  |  |  |  |  |
| Migraine + ≥ 1 other chronic condition | 202 (0.1) | 1.88 (1.61-2.20) | 1.84 (1.57-2.16) | 0.29 (-0.14,0.69) | 16.0 (-8.83,34.06) |
| Migraine alone | 59 (0.1) | 1.18 (0.91-1.54) | 1.20 (0.92-1.56) |  |  |
| Other chronic condition(s) alone | 996 (0.1) | 1.38 (1.25-1.52) | 1.35 (1.22-1.49) |  |  |
| No migraine/other chronic condition | 697 (0.1) | 1.00 (ref) | 1.00 (ref) |  |  |
| **Hysterectomy** |  |  |  |  |  |
| Migraine + ≥ 1 other chronic condition | 309 (0.2) | 1.28 (1.13-1.44) | 1.34 (1.19-1.52) | 0.01 (-0.26,0.27) | 1.10 (-21.02,18.38) |
| Migraine alone | 126 (0.2) | 1.12 (0.93-1.34) | 1.17 (0.98-1.41) |  |  |
| Other chronic condition(s) alone | 1837 (0.2) | 1.13 (1.06-1.21) | 1.16 (1.08-1.24) |  |  |
| No migraine/other chronic condition | 1572 (0.1) | 1.00 (ref) | 1.00 (ref) |  |  |
| **Maternal ICU admission** |  |  |  |  |  |
| Migraine + ≥ 1 other chronic condition | 813 (0.5) | 2.07 (1.91-2.25) | 2.04 (1.89-2.21) | 0.37 (0.14,0.58) | 17.9 (7.12,27.04) |
| Migraine alone | 201 (0.2) | 1.11 (0.96-1.28) | 1.12 (0.97-1.29) |  |  |
| Other chronic condition(s) alone | 4152 (0.3) | 1.58 (1.51-1.66) | 1.56 (1.48-1.64) |  |  |
| No migraine/other chronic condition | 2524 (0.2) | 1.00 (ref) | 1.00 (ref) |  |  |
| **Surgical complications** |  |  |  |  |  |
| Migraine + ≥ 1 other chronic condition | 259 (0.1) | 1.51 (1.32-1.73) | 1.48 (1.29-1.69) | 0.03 (-0.30,0.33) | 1.96 (-22.1,20.3) |
| Migraine alone | 94 (0.1) | 1.18 (0.95-1.45) | 1.18 (0.96-1.46) |  |  |
| Other chronic condition(s) alone | 1490 (0.1) | 1.29 (1.20-1.40) | 1.27 (1.17-1.37) |  |  |
| No migraine/other chronic condition | 1115 (0.1) | 1.00 (ref) | 1.00 (ref) |  |  |
| **Other** |  |  |  |  |  |
| Migraine + ≥ 1 other chronic condition | 456 (0.3) | 1.72 (1.55-1.91) | 1.69 (1.52-1.88) | 0.24 (-0.02,0.48) | 14.1 (-1.57,26.7) |
| Migraine alone | 132 (0.2) | 1.08 (0.89-1.30) | 1.07 (0.89-1.29) |  |  |
| Other chronic condition(s) alone | 2458 (0.2) | 1.40 (1.32-1.49) | 1.38 (1.30-1.47) |  |  |
| No migraine/other chronic condition | 1686 (0.1) | 1.00 (ref) | 1.00 (ref) |  |  |
| **Maternal mortality (≤ 42 days postpartum)** |  |  |  |  |  |
| Migraine + ≥ 1 other chronic condition | 16 (0.00) | 1.53 (0.89-2.64) | 1.42 (0.83-2.45) | -1.05 (-2.93,0.15) | -74.0 (-262.9, -5.71) |
| Migraine alone | 6 (0.00) | 1.23 (0.53-2.83) | 1.24 (0.54-2.85) |  |  |
| Other chronic condition(s) alone | 167 (0.00) | 2.38 (1.79-3.15) | 2.23 (1.68-2.97) |  |  |
| No migraine/other chronic condition | 68 (0.00) | 1.00 (ref) | 1.00 (ref) |  |  |

Abbreviations: aAP = adjusted attributable proportion due to interaction; aRERI = relative excess risk due to interaction; aRR = adjusted relative risk; CI = confidence interval; DIC = disseminated intravascular coagulation; HELLP = hemolysis, elevated liver enzymes, and low platelets syndrome; ICU = intensive care unit; RR = relative risk; SMM-M = severe maternal morbidity or mortality; SPE = severe preeclampsia.

Note: aRR, aRERI, and aAP adjusted for age, year of conception, parity, neighbourhood income quintile, rural residence, immigrant/refugee status, and recent history of interpersonal violence.

**Table S12.** Additive interaction between migraine in the 5 years before conception and any other chronic condition on the risks of individual indicators of SNM.

| Risk Factor | N (%) with outcome | RR  (95% CI) | aRR  (95% CI) | aRERI  (95% CI) | aAP (%)  (95% CI) |  |
| --- | --- | --- | --- | --- | --- | --- |
| **Gestational age <32 weeks** |  |  |  |  |  |  |
| Migraine + ≥ 1 other chronic condition | 1210 (1.1) | 1.52 (1.43-1.62) | 1.52 (1.42-1.61) | 0.11 (-0.04,0.25) | 7.09 (-2.62,15.6) |  |
| Migraine alone | 423 (0.8) | 1.10 (0.99-1.21) | 1.11 (1.00-1.22) |  |  |  |
| Other chronic condition(s) alone | 7107 (0.9) | 1.31 (1.27-1.36) | 1.30 (1.26-1.35) |  |  |  |
| No migraine/other chronic condition | 5487 (0.7) | 1.00 (ref) | 1.00 (ref) |  |  |  |
| **Birthweight < 1500 g** |  |  |  |  |  |  |
| Migraine + ≥ 1 other chronic condition | 1064 (1.0) | 1.54 (1.44-1.65) | 1.54 (1.44-1.64) | 0.05 (-0.11,0.20) | 3.35 (-7.32,12.7)B |  |
| Migraine alone | 396 (0.7) | 1.17 (1.06-1.30) | 1.19 (1.07-1.31) |  |  |  |
| Other chronic condition(s) alone | 6163 (0.8) | 1.31 (1.26-1.36) | 1.30 (1.25-1.35) |  |  |  |
| No migraine/other chronic condition | 4781 (0.6) | 1.00 (ref) | 1.00 (ref) |  |  |  |
| **Birth trauma** |  |  |  |  |  |  |
| Migraine + ≥ 1 other chronic condition | 288 (0.3) | 1.21 (1.06-1.37) | 1.19 (1.05-1.35) | -0.04 (-0.28,0.19) | -3.14 (-2.54,13.9) |  |
| Migraine alone | 104 (0.2) | 0.88 (0.72-1.07) | 0.88 (0.72-1.07) |  |  |  |
| Other chronic condition(s) alone | 2218 (0.3) | 1.36 (1.28-1.45) | 1.36 (1.27-1.45) |  |  |  |
| No migraine/other chronic condition | 1670 (0.2) | 1.00 (ref) | 1.00 (ref) |  |  |  |
| **Necrotizing enterocolitis** |  |  |  |  |  |  |
| Migraine + ≥ 1 other chronic condition | 148 (0.1) | 1.53 (1.28-1.82) | 1.50 (1.26-1.80) | -0.49 (-0.97, -0.07) | -3.26 (-70.9, -5.19) |  |
| Migraine alone | 71 (0.1) | 1.47 (1.15-1.88) | 1.49 (1.17-1.90) |  |  |  |
| Other chronic condition(s) alone | 1015 (0.1) | 1.53 (1.39-1.69) | 1.51 (1.37-1.66) |  |  |  |
| No migraine/other chronic condition | 679 (0.1) | 1.00 (ref) | 1.00 (ref) |  |  |  |
| **Seizures** |  |  |  |  |  |  |
| Migraine + ≥ 1 other chronic condition | 327 (0.3) | 1.68 (1.49-1.90) | 1.68 (1.49-1.90) | 0.08 (-0.22,0.36) | 4.89 (-14.4,20.0) |  |
| Migraine alone | 110 (0.2) | 1.14 (0.94-1.38) | 1.14 (0.94-1.38) |  |  |  |
| Other chronic condition(s) alone | 1932 (0.3) | 1.45 (1.36-1.56) | 1.46 (1.36-1.56) |  |  |  |
| No migraine/other chronic condition | 1359 (0.2) | 1.00 (ref) | 1.00 (ref) |  |  |  |
| **Intraventricular haemorrhage (grade 2, 3, or 4)** | | | | | |  |
| Migraine + ≥ 1 other chronic condition | 129 (0.1) | 1.48 (1.22-1.79) | 1.46 (1.21-1.77) | 0.04 (-0.41,0.43) | 2.53 (-31.4,25.4) |  |
| Migraine alone | 43 (0.1) | 1.00 (0.73-1.36) | 1.00 (0.73-1.36) |  |  |  |
| Other chronic condition(s) alone | 858 (0.1) | 1.44 (1.30-1.60) | 1.43 (1.28-1.58) |  |  |  |
| No migraine/other chronic condition | 610 (0.1) | 1.00 (ref) | 1.00 (ref) |  |  |  |
| **Cerebral infarction** |  |  |  |  |  |  |
| Migraine + ≥ 1 other chronic condition | 18 (0.0) | 1.31 (0.79-2.17) | Did not converge | Did not converge | Did not converge |  |
| Migraine alone | 6 (0.0) | 0.88 (0.39-2.01) | Did not converge |  |  |  |
| Other chronic condition(s) alone | 120 (0.0) | 1.28 (0.98-1.68) | Did not converge |  |  |  |
| No migraine/other chronic condition | 96 (0.0) | 1.00 (ref) | Did not converge |  |  |  |
| **Periventricular leukomalacia** |  |  |  |  |  |  |
| Migraine + ≥ 1 other chronic condition | 25 (0.0) | 1.38 (0.90-2.12) | Did not converge | Did not converge | Did not converge |  |
| Migraine alone | 8 (0.0) | 0.89 (0.43-1.81) | Did not converge |  |  |  |
| Other chronic condition(s) alone | 169 (0.0) | 1.37 (1.09-1.72) | Did not converge |  |  |  |
| No migraine/other chronic condition | 127 (0.0) | 1.00 (ref) | Did not converge |  |  |  |
| **Neonatal encephalopathy** |  |  |  |  |  |  |
| Migraine + ≥ 1 other chronic condition | 311 (0.3) | 1.30 (1.15-1.47) | 1.32 (1.17-1.49) | -0.09 (-0.36,0.15) | -6.96 (-2.91,10.4) |  |
| Migraine alone | 134 (0.2) | 1.13 (0.95-1.35) | 1.14 (0.95-1.36) |  |  |  |
| Other chronic condition(s) alone | 2064 (0.3) | 1.27 (1.19-1.35) | 1.27 (1.19-1.36) |  |  |  |
| No migraine/other chronic condition | 1673 (0.2) | 1.00 (ref) | 1.00 (ref) |  |  |  |
| **Infection** |  |  |  |  |  |  |
| Migraine + ≥ 1 other chronic condition | 1503 (1.4) | 1.35 (1.28-1.43) | 1.35 (1.27-1.42) | 0.12 (0.01,0.23) | 8.95 (0.34,16.6) |  |
| Migraine alone | 557 (1.0) | 1.01 (0.93-1.10) | 1.01 (0.93-1.10) |  |  |  |
| Other chronic condition(s) alone | 9220 (1.2) | 1.22 (1.18-1.26) | 1.22 (1.18-1.25) |  |  |  |
| No migraine/other chronic condition | 7755 (1.0) | 1.00 (ref) | 1.00 (ref) |  |  |  |
| **Respiratory distress syndrome** | | | | | |  |
| Migraine + ≥ 1 other chronic condition | 1941 (1.8) | 1.64 (1.56-1.72) | 1.63 (1.55-1.71) | 0.11 (-0.01,0.22) | 6.66 (-0.78,13.4) |  |
| Migraine alone | 654 (1.2) | 1.13 (1.04-1.22) | 1.14 (1.05-1.23) |  |  |  |
| Other chronic condition(s) alone | 11,258 (1.5) | 1.40 (1.36-1.44) | 1.38 (1.34-1.42) |  |  |  |
| No migraine/other chronic condition | 8184 (1.1) | 1.00 (ref) | 1.00 (ref) |  |  |  |
| **Pneumonia** |  |  |  |  |  |  |
| Migraine + ≥ 1 other chronic condition | 334 (0.3) | 1.46 (1.29-1.64) | 1.42 (1.26-1.60) | 0.00 (-0.27,0.25) | 0.27 (-20.2,16.4) |  |
| Migraine alone | 129 (0.2) | 1.13 (0.95-1.35) | 1.13 (0.94-1.35) |  |  |  |
| Other chronic condition(s) alone | 2046 (0.3) | 1.31 (1.23-1.40) | 1.29 (1.21-1.38) |  |  |  |
| No migraine/other chronic condition | 1605 (0.2) | 1.00 (ref) | 1.00 (ref) |  |  |  |
| **Chronic respiratory conditions originating in the perinatal period** | | | | | |  |
| Migraine + ≥ 1 other chronic condition | 229 (0.2) | 1.55 (1.34-1.79) | 1.54 (1.33-1.78) | 0.21 (-0.13,0.05) | 13.7 (-9.43,30.9) |  |
| Migraine alone | 77 (0.1) | 1.05 (0.84-1.33) | 1.06 (0.84-1.34) |  |  |  |
| Other chronic condition(s) alone | 1290 (0.2) | 1.28 (1.18-1.39) | 1.27 (1.17-1.38) |  |  |  |
| No migraine/other chronic condition | 1034 (0.1) | 1.00 (ref) | 1.00 (ref) |  |  |  |
| **Other respiratory** |  |  |  |  |  |  |
| Migraine + ≥ 1 other chronic condition | 1789 (1.6) | 1.50 (1.43-1.58) | 1.50 (1.42-1.58) | 0.09 (-0.03,0.20) | 5.75 (-2.13,12.8) |  |
| Migraine alone | 647 (1.2) | 1.10 (1.01-1.19) | 1.10 (1.02-1.19) |  |  |  |
| Other chronic condition(s) alone | 10,756 (1.4) | 1.32 (1.29-1.36) | 1.31 (1.27-1.35) |  |  |  |
| No migraine/other chronic condition | 8326 (1.1) | 1.00 (ref) | 1.00 (ref) |  |  |  |
| **Perinatal intestinal perforation** | | | |  |  |  |
| Migraine + ≥ 1 other chronic condition | 43 (0.0) | 1.54 (1.10-2.14) | Did not converge | Did not converge | Did not converge |  |
| Migraine alone | 14 (0.0) | 1.01 (0.59-1.73) | Did not converge |  |  |  |
| Other chronic condition(s) alone | 250 (0.0) | 1.31 (1.09-1.58) | Did not converge |  |  |  |
| No migraine/other chronic condition | 196 (0.0) | 1.00 (ref) | Did not converge |  |  |  |
| **Retinopathy of prematurity** |  |  |  |  |  |  |
| Migraine + ≥ 1 other chronic condition | 367 (0.3) | 1.58 (1.41-1.76) | 1.58 (1.41-1.77) | 0.11 (-0.16,0.37) | 6.98 (-11.2,21.4) |  |
| Migraine alone | 126 (0.2) | 1.09 (0.91-1.31) | 1.11 (0.93-1.33) |  |  |  |
| Other chronic condition(s) alone | 2175 (0.3) | 1.37 (1.28-1.46) | 1.36 (1.28-1.45) |  |  |  |
| No migraine/other chronic condition | 1627 (0.2) | 1.00 (ref) | 1.00 (ref) |  |  |  |
| **Ventilatory support** |  |  |  |  |  |  |
| Migraine + ≥ 1 other chronic condition | 6098 (5.5) | 1.43 (1.39-1.47) | 1.45 (1.41-1.49) | 0.04 (-0.02,0.10) | 2.51 (-1.74,6.53) |  |
| Migraine alone | 2284 (4.2) | 1.09 (1.04-1.13) | 1.09 (1.05-1.14) |  |  |  |
| Other chronic condition(s) alone | 38,077 (5.1) | 1.31 (1.29-1.33) | 1.31 (1.30-1.33) |  |  |  |
| No migraine/other chronic condition | 29,673 (3.8) | 1.00 (ref) | 1.00 (ref) |  |  |  |
| **Pneumothorax requiring intercostal catheter** | | | |  |  |  |
| Migraine + ≥ 1 other chronic condition | 215 (0.2) | 1.49 (1.29-1.73) | 1.48 (1.28-1.71) | 0.06 (-0.30,0.38) | 3.84 (-22.5,23.5) |  |
| Migraine alone | 91 (0.2) | 1.27 (1.03-1.58) | 1.28 (1.03-1.59) |  |  |  |
| Other chronic condition(s) alone | 1136 (0.2) | 1.16 (1.06-1.26) | 1.14 (1.05-1.25) |  |  |  |
| No migraine/other chronic condition | 1007 (0.1) | 1.00 (ref) | 1.00 (ref) |  |  |  |
| **Any body cavity surgical procedure** | | | |  |  |  |
| Migraine + ≥ 1 other chronic condition | 338 (0.3) | 1.25 (1.11-1.40) | 1.23 (1.10-1.38) | -0.08 (-0.32,0.14) | -6.70 (-28.2,10.3) |  |
| Migraine alone | 147 (0.3) | 1.09 (0.92-1.29) | 1.09 (0.93-1.29) |  |  |  |
| Other chronic condition(s) alone | 2280 (0.3) | 1.23 (1.16-1.31) | 1.22 (1.15-1.30) |  |  |  |
| No migraine/other chronic condition | 1896 (0.3) | 1.00 (ref) | 1.00 (ref) |  |  |  |
| **Resuscitation by intubation and/or chest compressions** | | | | |  |  |
| Migraine + ≥ 1 other chronic condition | 286 (0.3) | 1.37 (1.21-1.55) | 1.35 (1.19-1.54) | -0.05 (-0.33,0.22) | -3.39 (-2.65,1.46) |  |
| Migraine alone | 121 (0.2) | 1.17 (0.97-1.41) | 1.17 (0.98-1.41) |  |  |  |
| Other chronic condition(s) alone | 1770 (0.2) | 1.24 (1.16-1.33) | 1.22 (1.14-1.31) |  |  |  |
| No migraine/other chronic condition | 1461 (0.2) | 1.00 (ref) | 1.00 (ref) |  |  |  |
| **Central venous or arterial catheter insertion** | | | |  |  |  |
| Migraine + ≥ 1 other chronic condition | 2036 (1.9) | 1.54 (1.47-1.62) | 1.53 (1.46-1.61) | 0.01 (-0.10,0.12) | 0.62 (-7.01,7.53) |  |
| Migraine alone | 698 (1.3) | 1.08 (1.00-1.16) | 1.09 (1.01-1.17) |  |  |  |
| Other chronic condition(s) alone | 12,649 (1.7) | 1.41 (1.37-1.45) | 1.39 (1.35-1.43) |  |  |  |
| No migraine/other chronic condition | 9173 (1.2) | 1.00 (ref) | 1.00 (ref) |  |  |  |
| **Administration of any intravenous fluid** | | | |  |  |  |
| Migraine + ≥ 1 other chronic condition | 2734 (2.5) | 1.52 (1.46-1.59) | 1.52 (1.46-1.58) | 0.07 (-0.02,0.16) | 4.70 (-1.68,10.6) |  |
| Migraine alone | 975 (1.8) | 1.11 (1.04-1.18) | 1.12 (1.05-1.20) |  |  |  |
| Other chronic condition(s) alone | 16,358 (2.2) | 1.34 (1.31-1.37) | 1.33 (1.30-1.36) |  |  |  |
| No migraine/other chronic condition | 12,425 (1.6) | 1.00 (ref) | 1.00 (ref) |  |  |  |
| **Transfusion of red blood cells or a blood product** | | | |  |  |  |
| Migraine + ≥ 1 other chronic condition | [masked] | 1.89 (1.00-3.56) | Did not converge | Did not converge | Did not converge |  |
| Migraine alone | [masked] | 0.63 (0.15-2.60) | Did not converge |  |  |  |
| Other chronic condition(s) alone | 47 (0.0) | 1.07 (0.71-1.62) | Did not converge |  |  |  |
| No migraine/other chronic condition | 45 (0.0) | 1.00 (ref) | Did not converge |  |  |  |
| **Neonatal (<28 days) or in-hospital death** | | |  |  |  |  |
| Migraine + ≥ 1 other chronic condition | 258 (0.2) | 1.40 (1.22-1.60) | 1.36 (1.19-1.56) | 0.14 (-0.15,0.41) | 10.6 (-12.0,27.8) |  |
| Migraine alone | 93 (0.2) | 1.02 (0.83-1.26) | 1.02 (0.83-1.27) |  |  |  |
| Other chronic condition(s) alone | 1535 (0.2) | 1.22 (1.13-1.31) | 1.19 (1.11-1.29) |  |  |  |
| No migraine/other chronic condition | 1287 (0.2) | 1.00 (ref) | 1.00 (ref) |  |  |  |

Abbreviations: aAP = adjusted attributable proportion due to interaction; aRERI = adjusted relative excess risk due to interaction; aRR = adjusted relative risk; CI = confidence interval; RR = relative risk; SNM-M = severe neonatal morbidity or mortality.

Note: aRR, aRERI, and aAP adjusted for age, year of conception, parity, neighbourhood income quintile, rural residence, immigrant/refugee status, recent history of interpersonal violence, and fetal sex. Only crude estimates are reported for: cerebral infraction, periventricular leukomalacia, and perinatal intestinal perforation due to small n in the “migraine alone” subgroup, leading to model nonconvergence.


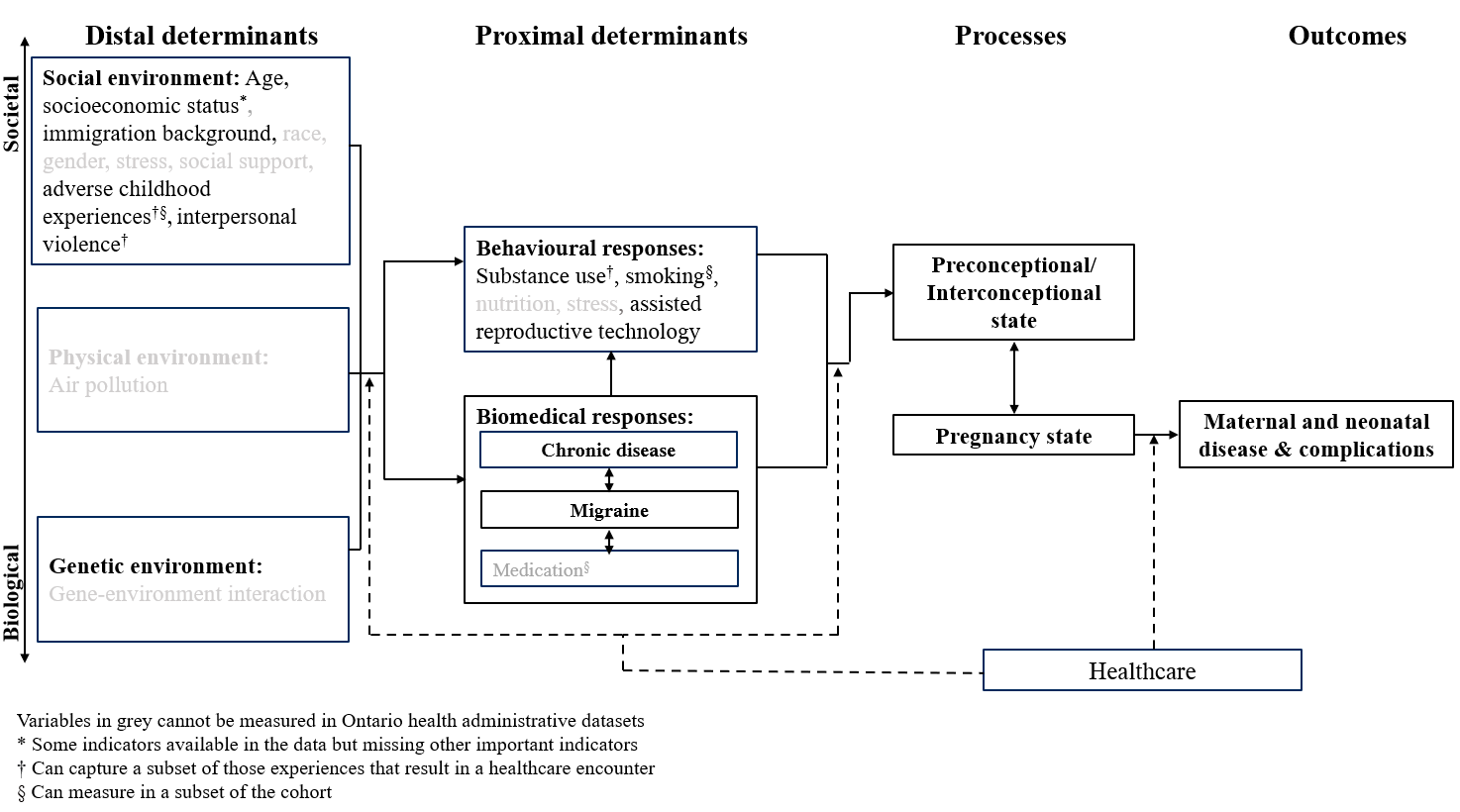


**Figure S1.** Conceptual framework of migraine, associated chronic diseases, and maternal and neonatal outcomes.

This conceptual framework builds on Dawn Misra’s Integrated Perinatal Health Framework (39).


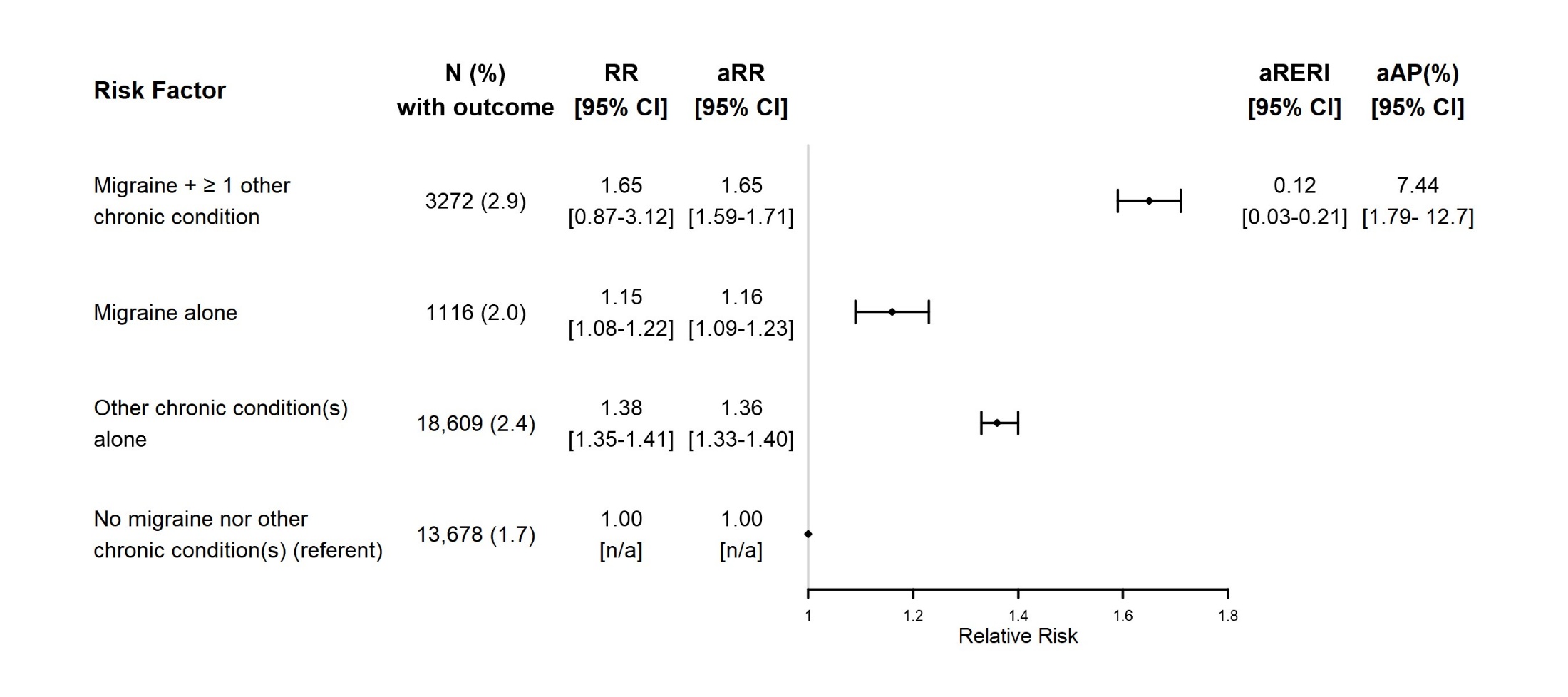


**Figure S2.** Additive interaction between migraine in the 5 years before conception and any other chronic condition on the risk of SMM-M among N=1,734,729 pregnancies ending in a livebirth or stillbirth and additionally adjusted for multiple birth status.

Abbreviations: aAP = adjusted attributable proportion due to interaction; aRERI = adjusted relative excess risk due to interaction; aRR = adjusted relative risk; CI = confidence interval; RR = relative risk; SMM-M = severe maternal morbidity or mortality; SNM-M = severe neonatal morbidity or mortality.

Note: aRR, aRERI, and aAP adjusted for age, year of conception, parity, neighbourhood income quintile, rural residence, immigrant/refugee status, and recent history of interpersonal violence, and multiple birth (e.g. twin or triplet) status.


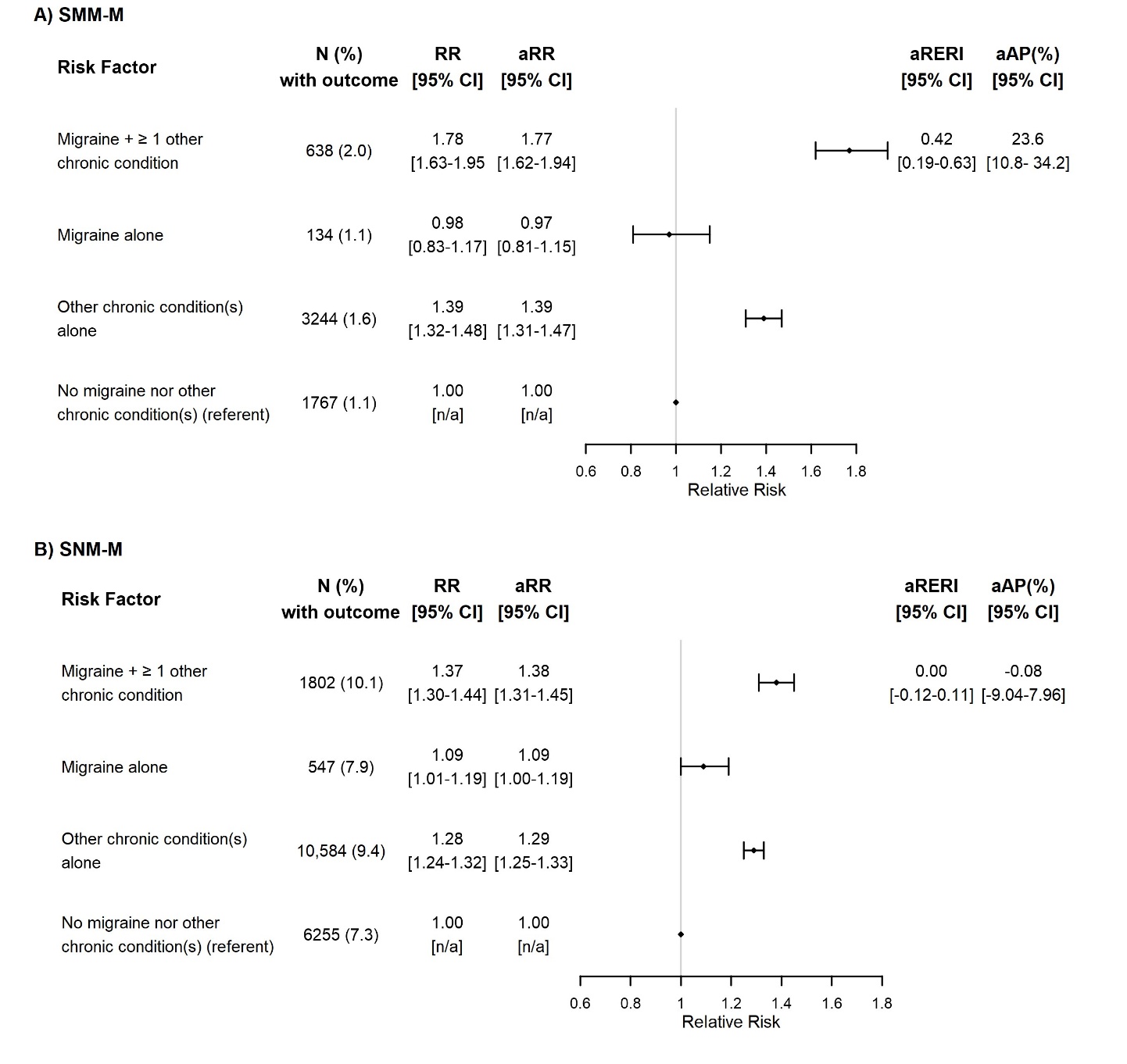


**Figure S3.** Additive interaction between migraine in the 5 years before conception and any other chronic condition on the risks of A) SMM-M and B) SNM-M among individuals born in Ontario and additionally adjusted for history of adverse childhood experiences.

Abbreviations: aAP = adjusted attributable proportion due to interaction; aRERI = adjusted relative excess risk due to interaction; aRR = adjusted relative risk; CI = confidence interval; RR = relative risk; SMM-M = severe maternal morbidity or mortality; SNM-M = severe neonatal morbidity or mortality.

Note: aRR, aRERI, and aAP adjusted for age, year of conception, parity, neighbourhood income quintile, rural residence, immigrant/refugee status, recent history of interpersonal violence, any adverse childhood experience, and (for SNM-M only) fetal sex.

# **References for supplemental material**

1. Austin PC. Using the standardized difference to compare the prevalence of a binary variable between two groups in observational research. *Commun Stat - Simul Comput.* 2009;38(6):1228-1234. doi:10.1080/03610910902859574
2. Statistics Canada. Table 13-10-0429-01 Live births and fetal deaths (stillbirths), by place of birth (hospital or non-hospital). Available at https://www150.statcan.gc.ca/t1/tbl1/en/tv.action?pid=1310042901
3. Varner CE, Park AL, Little D, Ray JG. Emergency department use by pregnant women in Ontario: a retrospective population-based cohort study. *cmajo.* 2020 Apr;8(2):E304–12.
4. Albanese CM, Bondy SJ, Lay C, Li Z, Guan J, Brown HK. Use of health administrative data to identify migraine in individuals with a recognized pregnancy: A validation study in Ontario, Canada. *Epidemiology.* 2025;36(5):599-605.
5. Gershon AS, Wang C, Guan J, Vasilevska-Ristovska J, Cicutto L, To T. Identifying patients with physician-diagnosed asthma in health administrative databases. *Can Respir J.* 2009;16(6):183-188. doi:10.1155/2009/963098
6. Tu K, Nieuwlaat R, Cheng SY, et al. Identifying patients with atrial fibrillation in administrative data. *Can J Cardiol.* 2016;32(12):1561-1565. doi:10.1016/j.cjca.2016.06.006
7. Zhu Y, Liu W, Li Y, Wang X, Winterstein AG. Prevalence of ADHD in publicly insured adults. *J Atten Disord.* 2018;22(2):182-190. doi:10.1177/1087054717698815
8. Weiss JA, Isaacs B, Diepstra H, et al. Health concerns and health Service utilization in a population cohort of young adults with autism spectrum disorder. *J Autism Dev Disord.* 2018;48(1):36-44. doi:10.1007/s10803-017-3292-0
9. Tu K, Campbell NR, Chen ZL, Cauch-Dudek KJ, McAlister FA. Accuracy of administrative databases in identifying patients with hypertension. *Open Med.* 2007;1(1):e18-e26
10. Philip G, Djerboua M, Carlone D, Flemming JA. Validation of a hierarchical algorithm to define chronic liver disease and cirrhosis etiology in administrative healthcare data. *PLoS One.* 2020;15(2):e0229218. doi:10.1371/journal.pone.0229218
11. Gershon AS, Wang C, Guan J, Vasilevska-Ristovska J, Cicutto L, To T. Identifying individuals with physician diagnosed COPD in health administrative databases. *COPD.* 2009;6(5):388-394. doi:10.1080/15412550903140865
12. Schultz SE, Rothwell DM, Chen Z, Tu K. Identifying cases of congestive heart failure from administrative data: a validation study using primary care patient records. *Chronic Dis Inj Can.* 2013;33(3):160-166.
13. Tu K, Mitiku T, Lee DS, Guo H, Tu JV. Validation of physician billing and hospitalization data to identify patients with ischemic heart disease using data from the Electronic Medical Record Administrative data Linked Database (EMRALD). *Can J Cardiol.* 2010;26(7):e225-e228. doi:10.1016/s0828-282x(10)70412-8
14. Hux JE, Ivis F, Flintoft V, Bica A. Diabetes in Ontario: determination of prevalence and incidence using a validated administrative data algorithm. *Diabetes Care.* 2002;25(3):512-516. doi:10.2337/diacare.25.3.512
15. Velez MP, Bougie O, Bahta L, et al. Mode of conception in patients with endometriosis and adverse pregnancy outcomes: a population-based cohort study. *Fertil Steril.* 2022;118(6):1090-1099. doi:10.1016/j.fertnstert.2022.09.015
16. Tu K, Wang M, Jaakkimainen RL, et al. Assessing the validity of using administrative data to identify patients with epilepsy. *Epilepsia.* 2014;55(2):335-343. doi:10.1111/epi.12506
17. Redelmeier DA, Zung JD, Thiruchelvam D, Tibshirani RJ. Fibromyalgia and the risk of a subsequent motor vehicle crash. *J Rheumatol.* 2015;42(8):1502-1510. doi:10.3899/jrheum.141315
18. Benchimol EI, Guttmann A, Mack DR, et al. Validation of international algorithms to identify adults with inflammatory bowel disease in health administrative data from Ontario, Canada.*J Clin Epidemiol.* 2014;67(8):887-896. doi:10.1016/j.jclinepi.2014.02.019
19. Goff SL, Feld A, Andrade SE, et al. Administrative data used to identify patients with irritable bowel syndrome. *J Clin Epidemiol.* 2008;61(6):617-621. doi:10.1016/j.jclinepi.2007.07.013
20. Wong JJ, Côté P, Tricco AC, Watson T, Rosella LC. Assessing the validity of health administrative data compared to population health survey data for the measurement of low back pain. *Pain.* 2021;162(1):219-226. doi:10.1097/j.pain.0000000000002003
21. Institute for Clinical Evaluative Sciences (ICES). Mental Health and Addiction Program (MHAP) Framework: Guideline for incorporating mental health and addictions into health service research using ICES administrative data sources. Toronto, ON: ICES; 2022.
22. Widdifield J, Ivers NM, Young J, et al. Development and validation of an administrative data algorithm to estimate the disease burden and epidemiology of multiple sclerosis in Ontario, Canada. *Mult Scler.* 2015;21(8):1045-1054. doi:10.1177/1352458514556303
23. Austin PC, Daly PA, Tu JV. A multicenter study of the coding accuracy of hospital discharge administrative data for patients admitted to cardiac care units in Ontario. *Am Heart J.* 2002;144(2):290-296. doi:10.1067/mhj.2002.123839
24. Kuhle S, Kirk SF, Ohinmaa A, Veugelers PJ. Comparison of ICD code-based diagnosis of obesity with measured obesity in children and the implications for health care cost estimates. *BMC Med Res Methodol.* 2011;11:173. Published 2011 Dec 21. doi:10.1186/1471-2288-11-173
25. Clemens KK, Reid JN, Shariff SZ, Welk B. Validity of hospital codes for obesity in Ontario, Canada. *Can J Diabetes.* 2021;45(3):243-248.e4. doi:10.1016/j.jcjd.2020.08.106
26. Kendzerska T, van Walraven C, McIsaac DI, Povitz M, Mulpuru S, Lima I, Talarico R, Aaron SD, Reisman W, Gershon AS. Case-ascertainment models to identify adults with obstructive sleep apnea using health administrative data: Internal and external validation. *Clin Epidemiol.* 2021; 13:453-67. https://doi.org/10.2147/CLEP.S308852
27. Widdifield J, Jaakkimainen RL, Gatley JM, et al. Validation of canadian health administrative data algorithms for estimating trends in the incidence and prevalence of osteoarthritis. *Osteoarthr Cartil Open.* 2020;2(4):100115. Published 2020 Nov 4. doi:10.1016/j.ocarto.2020.100115
28. Eder L, Widdifield J, Rosen CF, Gladman DD, Alhusayen R, Paterson M, Cheng S, Jabbari S,Campbell W, Bernatsky S, Tu K. Accuracy of Canadian administrative health data in identifying patients with psoriasis and psoriatic arthritis using primary care medical records as the reference standard [abstract]. *Arthritis Rheumatol.* 2017; 69 (suppl 10)
29. Widdifield J, Bombardier C, Bernatsky S, et al. An administrative data validation study of the accuracy of algorithms for identifying rheumatoid arthritis: the influence of the reference standard on algorithm performance. *BMC Musculoskelet Disord.* 2014;15:216. Published 2014 Jun 23. doi:10.1186/1471-2474-15-216
30. Oliva L, Horlick E, Wang B, Huszti E, Hall R, Abrahamyan L. Developing a random forest algorithm to identify patent foramen ovale and atrial septal defects in Ontario administrative databases. *BMC Med Inform Decis Mak.* 2022;22(1):93. Published 2022 Apr 6. doi:10.1186/s12911-022-01837-2
31. Saunders NR, Gandhi S, Chen S, et al. Health care use and costs of children, adolescents, and young adults with somatic symptom and related disorders. *JAMA Netw Open.* 2020;3(7):e2011295. Published 2020 Jul 1. doi:10.1001/jamanetworkopen.2020.11295
32. Tu K, Wang M, Young J, et al. Validity of administrative data for identifying patients who have had a stroke or transient ischemic attack using EMRALD as a reference standard. *Can J Cardiol.* 2013;29(11):1388-1394. doi:10.1016/j.cjca.2013.07.676
33. Bernatsky S, Joseph L, Pineau CA, Tamblyn R, Feldman DE, Clarke AE. A population-based assessment of systemic lupus erythematosus incidence and prevalence--results and implications of using administrative data for epidemiological studies. *Rheumatology* (Oxford). 2007;46(12):1814-1818. doi:10.1093/rheumatology/kem233
34. Frost L, Vestergaard P, Mosekilde L. Hyperthyroidism and risk of atrial fibrillation or flutter: a population-based study [published correction appears in Arch Intern Med. 2005 Feb 14;165(3):307]. *Arch Intern Med.* 2004;164(15):1675-1678. doi:10.1001/archinte.164.15.1675
35. Hedegaard H, Johnson RL, Warner M, et al. Proposed framework for presenting injury data using the International Classification of Diseases, Tenth Revision, Clinician Modification (ICD-10-CM) diagnosis codes. *Natl Health Stat Rep.* 2016;22(89):1–20.
36. Le A, Lelli DA, Van Katwyk S, Hogan D, Thavorn K, Tse D. Dizziness at a Canadian tertiary care hospital: a cost-of-illness study. *J Otolaryngol Head Neck Surg*. 2019;48(1):5. doi:10.1186/s40463-019-0328-9
37. Felitti VJ, Anda RF, Nordenberg D, Williamson DF, Spitz AM, Edwards V, et al. Relationship of childhood abuse and household Dysfunction to many of the leading causes of death in adults*. Am J Prev Med.* 1998 May;14(4):245–58.
38. Fox MP, Fink A, Lash TL. Quantitative bias analysis spreadsheet [Microsoft Excel worksheet]. 2007. Available at: https://sites.google.com/site/biasanalysis. Accessed November 15, 2025.
39. Misra DP, Guyer B, Allston A. Integrated perinatal health framework. A multiple determinants model with a life span approach. *Am J Prev Med.* 2003;25(1):65-75. doi:10.1016/s0749-3797(03)00090-4
